# Who is placed, heard, and seen? Gendered opportunity pathways in Ghanaian senior high school choirs and marching bands

**DOI:** 10.3389/fpsyg.2026.1913572

**Published:** 2026-08-12

**Authors:** John-Doe Dordzro

**Affiliations:** Department of Music and Dance, University of Cape Coast, Cape Coast, Ghana

**Keywords:** educational equity, gendered opportunity pathways, Ghanaian music education, identity safety, instrument allocation, musical self-efficacy, rehearsal discourse, student leadership

## Abstract

Choirs and marching bands are important sites of musical learning, school representation, and youth leadership in Ghanaian senior high schools. Yet little is known about how gendered opportunity structures shape students’ access to instruments and roles, rehearsal experiences, leadership opportunities, and psychosocial participation. This convergent mixed-methods study examined gendered opportunity pathways in seven senior high schools in the Central Region of Ghana. Data comprised a survey of 236 ensemble participants, seven director interviews, seven student focus groups with 42 students, structured observations of 14 choir and marching band rehearsals, and a document audit of one performance cycle per school. Survey measures assessed belonging, music self-efficacy, stereotype experiences, identity safety, and intention to continue. The psychosocial scales were developed specifically for this study, informed by prior literature, and supported by acceptable internal consistency and preliminary factor-analytic evidence. Overall, 61.9% of students obtained their first-choice instrument or role, while 38.1% did not. First-choice allocation varied descriptively by gender, but the full-category association was not statistically significant. Private lessons and years of music experience were associated with higher odds of receiving first-choice allocation, while home instrument access approached conventional significance. Leadership/visibility roles were significantly associated with gender, with boys more likely than girls to hold visible responsibilities during the audited performance cycle. Observations showed that marching band rehearsals involved more discipline correction and peer teasing than choir rehearsals, and that feedback in band was more often directed towards boys. Qualitative accounts indicated that resource scarcity, opaque selection criteria, gendered assumptions about physicality and authority, and peer teasing shaped students’ belonging and identity safety. Findings are interpreted as exploratory and associative. The study suggests that gender equity in school music ensembles requires attention not only to access, but also to allocation procedures, visibility opportunities, feedback practices, and identity-safe rehearsal climates.

## Introduction

Participation in school music ensembles can be a powerful developmental experience. Choirs and marching bands provide students with opportunities to build musical skill, peer belonging, self-confidence, public identity, and leadership capacity. Yet ensemble participation is not simply a matter of joining a group. It also involves how opportunities are distributed: who receives preferred instruments or voice parts, who is positioned visibly, who is invited to lead, whose mistakes are corrected constructively, and whose musical contribution is publicly recognised. These everyday processes matter psychologically because they can support students’ belonging, music self-efficacy, identity safety, and intention to continue; they can also communicate exclusion, stereotype-based risk, or limited legitimacy (Goodenow, 1993a, 1993b; Bandura, 1997; Steele, 1997; Lamont, 2002; Walton and Cohen, 2007).

Gender is a recurring axis through which such opportunities become patterned. Research in music education has shown that instruments, voice parts, performance roles, and leadership behaviours can acquire gendered meanings, shaping who feels encouraged, authorised, or expected to occupy particular musical positions (Abeles and Porter, 1978; Green, 1997; Hallam et al., 2008; McClary, 1991; Sergeant and Himonides, 2014). More recent scholarship on music, gender, and social justice further suggests that gendered participation is often reproduced through everyday pedagogical routines, peer norms, and assumptions about authority, sound, confidence, and suitability rather than through explicit exclusion alone (Bradley, 2007; Gould, 2012; Hess, 2019; Koza, 1993; Werner et al., 2020). In ensemble settings, these routines may include assumptions about physical “fit,” differential access to leadership, gendered interpretations of mistakes, peer teasing, and opaque selection for solos or visible roles.

This study examines these issues in Ghanaian senior high schools. In Ghana, senior high school generally refers to the three-year upper secondary phase following junior high school, commonly enrolling adolescents in approximately Years 1–3 of senior secondary education. School music ensembles in this context often operate as co-curricular or extracurricular spaces connected to worship, public ceremonies, speech days, school competitions, parades, school identity, and student leadership. Ensemble directors may include trained music teachers, school staff with music responsibilities, or instructors with specialised ensemble experience, depending on school resources and staffing. These contextual details matter because music ensembles in Ghanaian senior high schools are not merely recreational clubs; they can function as public-facing institutions through which schools display discipline, artistry, identity, and prestige.

Choirs and marching bands also offer contrasting ensemble ecologies. Choirs often organise visibility through voice parts, solo singing, section leadership, and interpretive authority. Marching bands, by contrast, frequently involve public display, command structures, physical coordination, instrumental allocation, front-line positioning, and visible leadership roles such as drum major, band commander, or section leader. Comparing these ensemble forms within the same regional context therefore offers an opportunity to examine how gendered opportunity systems operate across different musical, social, and organisational settings. Although international research has shown that gendered expectations shape musical participation and instrument choice, little is known about how such dynamics operate in Ghanaian senior high school ensembles, particularly across choir and marching band settings. This gap matters because ensembles function not only as musical groups but also as systems of allocation, recognition, and authority. In resource-constrained school contexts, decisions about who receives preferred instruments or parts, who is invited to lead, and how mistakes are corrected may shape students’ belonging, identity safety, music self-efficacy, stereotype experiences, and intention to continue. This study therefore examines gendered opportunity pathways in senior high school music ensembles in the Central Region, Ghana, focusing on instrument/role allocation, rehearsal discourse, and student leadership/visibility.

## Literature review and conceptual framework

The literature informing this study is organised around four linked concerns: gendered allocation and constrained choice, rehearsal discourse and recognition climate, leadership/visibility as institutional recognition, and psychosocial mechanisms of participation. These strands are integrated into the study’s primary analytic framework: gendered opportunity pathways.

### Gendered allocation and constrained choice

Research on gender and music education has consistently shown that musical participation is shaped by the social meanings attached to instruments, voice, style, and performance roles. Early work on instrument stereotyping demonstrated that children and adolescents often associate particular instruments with masculinity or femininity, influencing what they perceive as appropriate or attainable (Abeles and Porter, 1978). Later studies showed that gendered patterns in instrument choice persist across many educational settings, although their form and intensity vary by culture, cohort, and institutional context (Hallam et al., 2008; Harrison, 2007; Sergeant and Himonides, 2014).

However, the language of “choice” can be misleading. In school ensembles, students’ preferences are often filtered through institutional needs, resource availability, teacher judgement, peer norms, and cost. This is especially important in contexts where instruments are limited, maintenance is expensive, uniforms or accessories require financial contribution, and directors must balance parts for public performance. Under such conditions, allocation becomes a form of opportunity distribution. Students’ prior access to instruments, private lessons, family support, and practice opportunities may function as cultural and educational advantages, shaping who can demonstrate readiness when ensemble roles are assigned (Bourdieu, 1986; Lareau, 2011). Who receives a preferred instrument, prominent voice part, front-line position, or visible role may shape later confidence, practice access, peer recognition, and leadership eligibility.

This point is particularly important in Ghanaian school music contexts. Dordzro’s work on Ghanaian basic school marching bands shows that instrument pathways are shaped by school traditions, availability, and teacher influence rather than by unconstrained preference alone (Dordzro, 2012, 2019). Broader African music education scholarship also reminds researchers that school music practices are embedded in local histories, community functions, performance traditions, institutional authority structures, and resource conditions (Akuno, 2005; Nzewi, 2003). The present study extends this concern to senior high school ensembles, where choir and marching band participation may carry heightened importance for school representation and youth leadership.

### Rehearsal discourse and recognition climate

If allocation determines where students enter an ensemble pathway, rehearsal discourse shapes how they experience that pathway. Rehearsals distribute powerful forms of recognition: instructional attention, correction, praise, permission to make mistakes, opportunities to speak, and invitations to demonstrate or lead. These practices may appear routine, but they communicate students’ relative status, competence, and belonging. Feedback research in education shows that the quality and framing of feedback influence learning, motivation, and perceived competence (Black and Wiliam, 1998; Hattie and Timperley, 2007). In ensemble settings, correction can be experienced as developmental when it focuses on musical improvement. However, correction may become identity-threatening when it is linked to gendered assumptions, such as the belief that girls are unsuited to particular instruments, boys should naturally produce a stronger sound, or leaders must display a particular kind of loudness or command.

Peer discourse also matters. Adolescents’ musical identities are negotiated within peer communities, where teasing, support, comparison, and senior–junior relations can shape participation. Communities of practice theory is useful here because ensemble learning involves movement from peripheral participation towards fuller recognition through practice, modelling, feedback, and social legitimacy (Lave and Wenger, 1991; Wenger, 1998).

### Leadership and visibility as institutional recognition

School ensembles are both instructional and public-facing organisations. They allocate not only learning opportunities but also visibility. Solos, featured parts, front-line positions, section leadership, first-chair status, drum major roles, band commander roles, and student conductor responsibilities are publicly legible signs of competence and trust. These roles can become forms of institutional recognition. Where selection criteria are explicit, transparent, and developmental, students may interpret non-selection as improvable. Where criteria are opaque, students may interpret exclusion through stereotypes, favouritism, or fixed assumptions about who naturally belongs in visible positions. Gender can operate subtly at this point. Leadership may be associated with loudness, command, physical presence, discipline, confidence, or public assertiveness. These traits may be read differently depending on whether they are performed by girls, boys, or gender-diverse students.

The concept of the hidden curriculum is useful for analysing these processes. The hidden curriculum refers to tacit lessons students learn through institutional routines, expectations, and informal norms rather than through formal instruction (Jackson, 1968; Margolis, 2001). In music ensembles, the hidden curriculum may teach students who is suited to particular instruments, whose leadership is legitimate, whose errors are treated as normal learning, and whose ambition invites ridicule. This does not require overt discrimination. Gendered opportunity pathways may be reproduced through ordinary placement decisions, rehearsal language, peer teasing, front positioning, and leadership invitations.

### Psychosocial mechanisms: belonging, identity safety, self-efficacy, and continuation

This study treats psychosocial constructs as context-sensitive experiences rather than fixed individual traits. Belonging refers to students’ sense of acceptance, fit, and membership within the ensemble community (Goodenow, 1993a, 1993b; Walton and Cohen, 2007). Identity safety refers to students’ perception that they can participate and be evaluated without being devalued based on gender or another social identity (Steele, 1997; Spencer et al., 1999; Davies et al., 2002; Steele et al., 2002). It is closely related to stereotype threat, which describes the risk of underperformance, withdrawal, or heightened self-monitoring when negative stereotypes become salient in evaluative settings (Steele, 1997; Spencer et al., 1999).

Music self-efficacy is also central. Bandura’s theory of self-efficacy emphasises that capability beliefs develop through mastery experiences, vicarious learning, social persuasion, and affective states (Bandura, 1997). Ensemble pathways directly implicate all four sources. Allocation affects access to mastery; rehearsal feedback shapes social persuasion; peer culture provides models and comparisons; and public performance or leadership roles intensify emotional and evaluative stakes. Expectancy-value theory further clarifies why these processes matter. Students are more likely to persist when they believe they can succeed and when participation feels valuable, meaningful, and identity-congruent (Eccles, 1994; Eccles and Wigfield, 2002). In senior high school contexts, intention to continue is especially important because participation may be constrained by academic workload, family expectations, timetabling, costs, and competing school responsibilities (Hallam et al., 2012).

Gender diversity adds another important inclusion concern. Gender identity, gender expression, and sexual orientation are distinct, although they may be socially conflated in school discourse and broader public debate. Research and policy guidance on measuring sex, gender identity, and sexual orientation emphasise the importance of conceptual clarity, privacy, and careful reporting, particularly when subgroup sizes are small or identities are socially sensitive (National Academies of Sciences, Engineering, and Medicine, 2022). In school music ensembles, inclusive practice therefore requires more than increasing girls’ participation in visible roles. It also requires attention to gendered comments, binary assumptions about instruments or voices, and rehearsal norms that may make participation feel conditional for students whose identities or expressions do not align with dominant expectations.

### Gendered opportunity pathways framework

Drawing these strands together, this study advances a gendered opportunity pathways framework. The framework provides the primary analytic lens for the study. It refers to patterned sequences through which students encounter allocation constraints, rehearsal discourse, leadership/visibility opportunities, and psychosocial participation experiences. It does not assume that gender causes outcomes in a simple or deterministic way. Rather, it examines how gendered meanings may cohere with institutional routines, resource conditions, and recognition practices.

The study uses a clear hierarchy of concepts. Gendered opportunity pathways serve as the primary analytic framework. Hidden curriculum and communities of practice explain the sociocultural mechanisms through which everyday ensemble routines teach students who belong, who are competent, and who may lead. Belonging, identity safety, self-efficacy, stereotype experiences, and intention to continue are treated as psychological mechanisms or participation experiences that may be shaped by these opportunity structures. Gender and inclusion provide the equity lens through which patterns of allocation, recognition, and rehearsal climate are interpreted.

**Figure 1:**
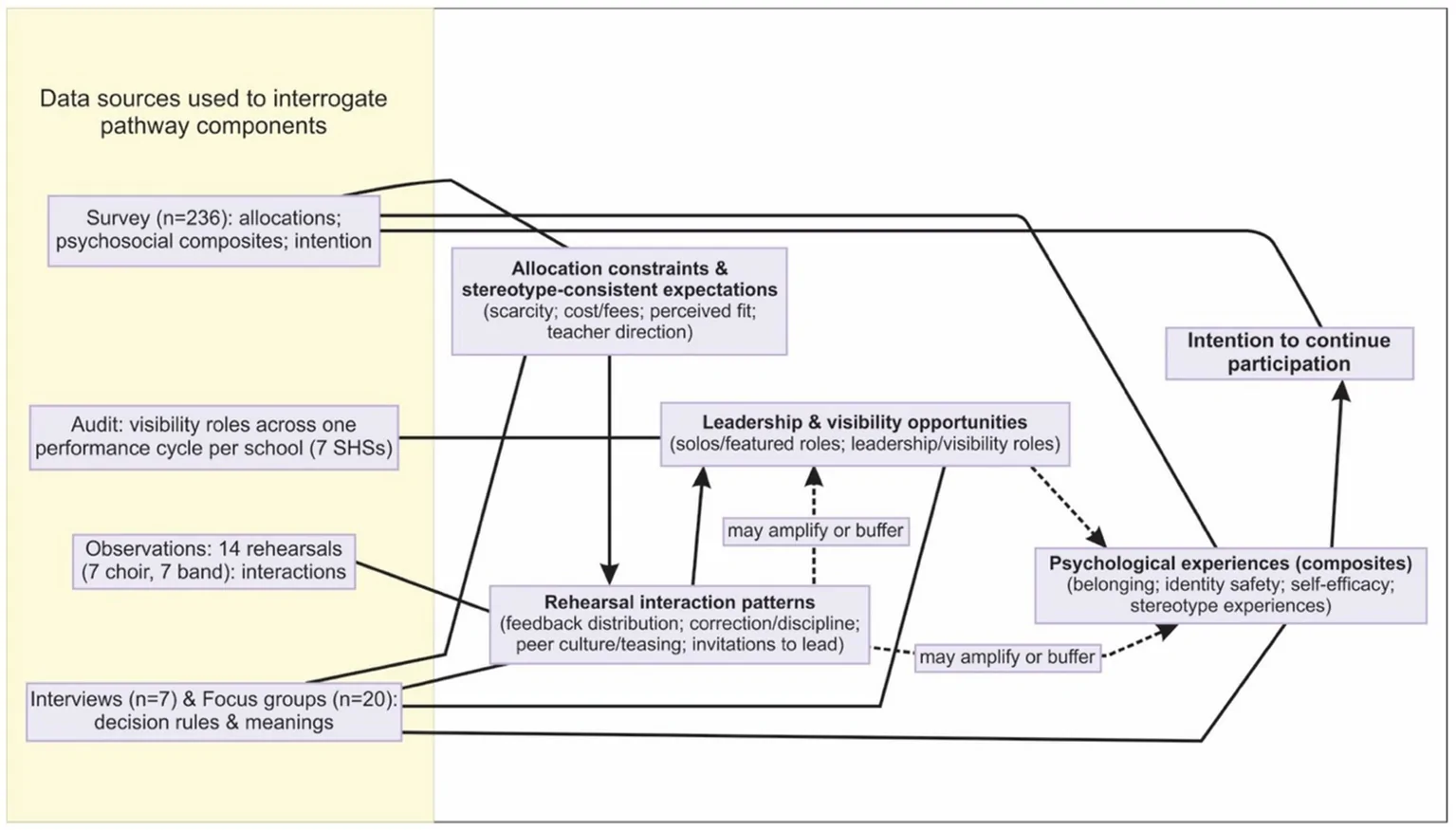
Conceptual model of gendered opportunity pathways in senior high school music ensembles.

This framework is especially appropriate for Ghanaian senior high school choirs and marching bands because these ensembles combine musical instruction, peer apprenticeship, public representation, and school prestige. It also avoids deficit framing. The study does not treat Ghanaian school ensembles as deficient versions of Global North models, but as culturally situated educational environments with their own performance functions, authority structures, and resource realities. Although the study foregrounds gender, students’ pathways may also be shaped by material access, private lesson opportunities, home instrument availability, disability or learning need disclosure, school type, and prior musical experience. The present study does not claim to conduct a full intersectional analysis. Rather, it identifies these factors as important contextual conditions and points toward future research that can examine how gender intersects with socioeconomic, disability, institutional, and regional differences in music participation (see Figure 1).

The model conceptualises ensemble participation as a sequence of linked opportunity processes: structural and material conditions; allocation and positioning; rehearsal discourse and peer climate; leadership and visibility; psychosocial participation experiences; and intention to continue. The model is interpretive and pathway-oriented rather than causal. This study addresses the following research questions: How are instrument/role allocations and leadership/visibility opportunities distributed amongst students in selected senior high school choir and marching band ensembles in the Central Region? What material, institutional, and perceived “fit” factors shape students’ non-attainment of preferred instruments or roles? What rehearsal discourse patterns, including feedback, correction, peer teasing, and leadership invitations, are observed in choir and marching band settings? How do student and director accounts explain, contextualise, or complicate survey, audit, and observation patterns in relation to belonging, identity safety, self-efficacy, stereotype experiences, and intention to continue?

## Methods

### Study design

This study employed a convergent mixed-methods design to examine gendered opportunity pathways in Ghanaian senior high school music ensembles. Quantitative and qualitative data were collected during the same phase of fieldwork, analysed separately, and then integrated through triangulation and joint display analysis. This design was appropriate because the study required both distributional evidence about allocation, leadership/visibility, rehearsal interaction, and psychosocial outcomes, and interpretive evidence about how students and ensemble directors understood these patterns in everyday school music practice. The quantitative strand comprised a student survey, a document audit of ensemble roles, and structured rehearsal observations. The qualitative strand comprised student focus groups, adult informant interviews with ensemble directors, and contextual field observations. Integration was achieved by aligning survey results, document audit indicators, observation counts, and qualitative excerpts to identify areas of convergence, complementarity, and expansion across data sources. The integrated analysis supported a pathway-oriented interpretation of ensemble participation rather than a causal explanation.

### Setting and school selection

The study was conducted in the Central Region of Ghana, a major educational region with senior high schools that maintain visible musical traditions, including choirs and marching bands. In Ghanaian senior high schools, these ensembles often contribute to worship, public ceremonies, speech days, parades, inter-school events, school identity, and student leadership.

Seven senior high schools were selected from the 124 senior high schools in the region using criterion-based sampling. Schools were eligible if they maintained both a school choir and a marching band during the fieldwork period. This criterion allowed comparison between two ensemble forms operating within broadly similar regional and administrative conditions. For confidentiality, participating schools are identified using anonymised codes, SHS1–SHS7. To protect institutional confidentiality, school-level characteristics such as ownership type, boarding/day status, gender composition, and mission/public/private status are not reported individually.

### Participants and data sources

Five data sources were used: a student survey, director interviews, student focus groups, structured rehearsal observations, and a document audit. The student survey was administered to 236 students in senior high school Years 1–3 who participated in choir, marching band, or both. Ensemble participation was coded as choir only, band only, or both choir and band. The sample included students from all seven participating schools and was relatively balanced by school and gender. Seven ensemble directors served as adult informants. They provided accounts of allocation procedures, rehearsal practices, leadership selection, visibility opportunities, resource constraints, peer climate, and inclusion-related concerns.

Student focus groups involved 42 students across seven focus group sessions. Students were purposively selected to ensure variation by year group, ensemble participation, gender, and leadership/visibility status. Focus groups were conducted in English, lasted approximately 30 min, were audio-recorded, and were transcribed verbatim before analysis. Two researchers conducted structured observations across 14 rehearsals, comprising seven choir rehearsals and seven marching band rehearsals. One choir rehearsal and one marching band rehearsal were observed in each participating school. Informal observations were also made during sectionals, pre-parade sessions, and other teaching weeks to support contextual understanding; however, only the structured, coded rehearsal observations are reported in the findings. A document audit was conducted for one performance cycle in each school. Available ensemble documents, including seating or positioning charts, leadership rosters, solo lists, and related performance records, were reviewed to map leadership/visibility opportunities and solo/featured roles.

### Survey instrument and measures

The student questionnaire included background variables, ensemble participation indicators, allocation/access items, rehearsal climate measures, and psychosocial scales. Unless otherwise stated, scale items used a five-point Likert-type response format ranging from 1, strongly disagree, to 5, strongly agree. Composite mean scores were computed for multi-item scales, with higher scores indicating stronger endorsement of the relevant construct. For stereotype-related measures, higher scores indicated greater reported experience of stereotype-related comments, cues, or assumptions in ensemble participation.

### Background and access variables

Background variables included school, senior high school year, gender identity, disability or learning need disclosure, private lessons, home instrument access, prior junior high school music participation, years of music experience, and a socioeconomic status proxy based on regular pocket money for school needs. Gender identity was reported using four categories: girl, boy, non-binary/gender-diverse, and prefer not to say. These categories were retained in descriptive reporting. Inferential analyses involving gender were interpreted cautiously where small cell sizes were present, particularly for non-binary/gender-diverse students and students who preferred not to disclose their gender. Prior music access variables included whether students had taken private lessons, whether they had access to an instrument at home, and whether they had participated in music during junior high school. Years of music experience were treated as a continuous variable in regression analysis.

### Ensemble participation

Ensemble participation was coded into three categories: choir only, band only, and both choir and band. This variable was used to examine differences in leadership/visibility opportunities and rehearsal climate across ensemble contexts. To examine allocation pathways, students were asked whether they received their first-choice instrument or role. First-choice allocation was coded as a binary variable: yes or no. Students who did not receive their first choice completed a multiple-response checklist indicating reasons for non-attainment. Response options included preferred instrument or role not available; cost or fees; teacher/director-directed placement; perceived “fit” expectations, including physicality, voice, or suitability; time or timetable constraints; and peer influence. Because multiple responses were allowed, percentages for reasons for non-attainment were calculated as the proportion of students who did not receive their first choice and were not expected to sum to 100%. Allocation satisfaction measured students’ satisfaction with their assigned instrument or role. Higher scores indicated greater satisfaction with allocation.

### Leadership/visibility and solo/featured roles

The document audit was used to operationalise public recognition and opportunity within ensembles. A student was coded as holding a leadership/visibility role if they held at least one of the following roles during the audited performance cycle: section leader or assistant section leader; first-chair or lead position; drum major or band commander; student conductor; public rehearsal leader; named soloist or featured performer; or other documented visible leadership or performance responsibility. A separate binary indicator captured whether a student held a solo/featured role. In addition, a leadership opportunity count was created to represent the number of leadership, demonstration, featured, or visible roles associated with a student.

### Rehearsal climate measures

The survey included measures of students’ perceptions of rehearsal climate. These included teacher feedback quality, peer encouragement, correction/criticism, inclusive discourse, and stereotype comments frequency. Teacher feedback quality captured students’ perceptions that feedback was constructive, specific, respectful, and useful for improvement. Peer encouragement assessed students’ perceptions of support, encouragement, and help from peers or senior students. Correction/criticism measured the extent to which rehearsal was experienced as correction-heavy, critical, or discipline-intensive. Inclusive discourse captured students’ perceptions that rehearsal talk and interaction were respectful, fair, and supportive of participation. Stereotype comments frequency measured students’ reports of comments, jokes, or assumptions linked to gendered expectations about instruments, voice, leadership, sound, strength, or suitability.

### Psychosocial measures and psychometric evidence

Psychosocial participation was measured using short composite scales developed specifically for this study and informed by prior work on belonging, self-efficacy, identity safety, stereotype threat, musical identity, recognition, and continuation intention. The scales were not adopted verbatim from a single existing instrument. Items were written to reflect Ghanaian senior high school choir and marching band participation, including rehearsal discourse, allocation, leadership, peer interaction, gendered “fit” assumptions, recognition, and continuation intention.

Composite mean scores were computed for belonging, music self-efficacy, stereotype experiences, identity safety, intention to continue, musical identity, and perceived recognition. Belonging measured students’ sense of acceptance, fit, and membership within the ensemble. Music self-efficacy measured confidence in learning, improving, and performing musically. Stereotype experiences assessed exposure to stereotype-related cues, comments, or assumptions. Identity safety measured whether students felt able to participate without being devalued because of gender or another social identity. Intention to continue assessed students’ reported likelihood or desire to continue participating in the ensemble. Musical identity captured the extent to which students viewed music participation as part of who they are. Perceived recognition measured the extent to which students felt noticed, valued, and acknowledged for their musical contributions.

Internal consistency was assessed using Cronbach’s alpha. The psychosocial scales demonstrated acceptable to strong reliability: belonging, *α* = 0.86; music self-efficacy, *α* = 0.82; stereotype experiences, *α* = 0.79; identity safety, *α* = 0.81; intention to continue, *α* = 0.84; musical identity, *α* = 0.83; and perceived recognition, *α* = 0.80. Because the scales were developed specifically for this study, additional construct-validity evidence was examined using exploratory and confirmatory factor analysis. Exploratory factor analysis used principal axis factoring with oblique rotation because the psychosocial constructs were expected to be correlated. The five-factor solution aligned with belonging, music self-efficacy, stereotype experiences, identity safety, and intention to continue. Most items loaded strongly on their intended factors, with primary loadings above 0.60 and limited problematic cross-loadings.

Confirmatory factor analysis supported the hypothesised five-factor model, χ^2^ = 331.42, *df* = 179*, CFI* = 0.942, *TLI* = 0.931, *RMSEA* = 0.060, and *SRMR* = 0.052. A one-factor comparison model showed poor fit, χ^2^ = 812.70, *df* = 189, *CFI* = 0.701, *TLI* = 0.668, *RMSEA* = 0.119, and *SRMR* = 0.104. These findings are interpreted as preliminary construct-validity evidence rather than final validation.

### Document audit procedures

The document audit was used to identify formal and public indicators of recognition within each ensemble. Available records for one performance cycle in each school were reviewed. These included leadership rosters, seating or positioning charts, solo or featured-role lists, performance orders, and related documents identifying students selected for visible responsibilities. Audit data were used to create binary indicators for any leadership/visibility role and any solo/featured role. Where possible, leadership opportunity counts were also derived to capture the number of visible or leadership-related opportunities associated with each student. Audit variables were reported in aggregate and, where ethically appropriate, by gender category. Small-cell reporting was handled cautiously to reduce re-identification risk.

### Structured rehearsal observations

Structured rehearsal observations were used to document interaction patterns relevant to learning, inclusion, recognition, and visibility. Fourteen rehearsals were observed: seven choir rehearsals and seven marching band rehearsals. One choir rehearsal and one marching band rehearsal were observed in each school. The observation protocol was developed specifically for this study rather than adopted wholesale from an existing instrument. It was informed by prior literature on classroom discourse, music rehearsal pedagogy, teacher feedback, student participation, peer interaction, stereotype threat, and identity safety. A study-specific protocol was necessary because the research questions focused on music-specific and equity-related practices, including role allocation, leadership invitations, gendered “fit” assumptions, corrective feedback, peer teasing, and feedback visibility by perceived gender.

Protocol development followed five stages: conceptual mapping to the research questions, review of relevant literature, development of operational categories, review for contextual relevance and appropriateness, and pilot testing before formal data collection. Pilot testing was used to refine category definitions and clarify ambiguous distinctions, including technical versus behavioural correction, general praise versus specific praise, routine participation versus leadership invitation, and general laughter versus peer teasing. The final protocol recorded event counts for technical corrective feedback, behaviour/discipline correction, specific teacher praise, talent or innate-ability statements, student-initiated musical suggestions, interruptions or over-talk incidents, leadership invitations, audible peer teasing, and feedback recipient gender.

Technical corrective feedback referred to teacher feedback directed at musical or performance improvement, including pitch, rhythm, tone, articulation, marching alignment, posture, breathing, instrumental technique, ensemble balance, timing, or related musical skills. Behaviour or discipline correction referred to feedback directed at conduct, attention, lateness, talking, movement, compliance, or general rehearsal behaviour. Peer teasing referred to audible peer comments, laughter, mimicry, joking, or ridicule directed at a student’s musical performance, instrument or role, voice, body, confidence, leadership, or perceived gendered suitability.

### Observation reliability

Two researchers carried out all structured observations using the predefined coding manual. To assess inter-rater reliability, a subset of observations was double-coded independently by both researchers. Inter-rater agreement was assessed using percentage agreement and Cohen’s kappa for categorical event codes. Intraclass correlation coefficients were also calculated for frequency-count variables. Disagreements were reviewed with reference to the coding manual, and final consensus codes were used in the analytic dataset. Inter-rater agreement was acceptable to strong across categories. Cohen’s kappa values ranged from *κ* = 0.65 for peer teasing incidents to *κ* = 0.88 for feedback recipient gender. Kappa values were *κ* = 0.81 for technical corrective feedback, *κ* = 0.77 for behaviour/discipline correction, *κ* = 0.79 for specific teacher praise, *κ* = 0.70 for talent/innate-ability statements, *κ* = 0.76 for student-initiated suggestions, *κ* = 0.68 for interruptions/over-talk, *κ* = 0.84 for leadership invitations, *κ* = 0.65 for peer teasing incidents, and *κ* = 0.88 for feedback recipient gender. Intraclass correlations ranged from *ICC* = 0.69 to *ICC* = 0.87, indicating moderate to good consistency for frequency counts.

### Feedback visibility by perceived gender

Observers recorded the perceived gender of students receiving feedback events using the categories girl, boy, and unclear/mixed. This coding was used only as an interactional indicator of who appeared to receive visible instructional attention during rehearsal. It was not treated as equivalent to students’ self-identified gender. Where perceived gender could not be determined with reasonable confidence, the event was coded as unclear or mixed. Feedback visibility results were interpreted descriptively because they captured observed interaction patterns during the sampled rehearsals, not all feedback events across the school year.

### Interviews and focus groups

Semi-structured protocols were used for student focus groups and director interviews. Focus groups were organised by school to reduce travel and scheduling disruption. Participants were purposively selected to include variation in year group, ensemble type, gender, and leadership/visibility status. The protocols explored instrument and role allocation processes; constraints affecting access to preferred roles; leadership and solo/featured-role selection; transparency of selection criteria; rehearsal norms; peer teasing and peer support; inclusive and exclusionary interaction patterns; gendered assumptions about instruments, voices, physicality, authority, and leadership; belonging; identity safety; self-efficacy; recognition; and continuation. Audio recordings were transcribed verbatim and anonymised.

### Quantitative analysis

Quantitative analyses were conducted using IBM SPSS Statistics. Descriptive statistics, reliability analysis, bivariate tests, regression models, mediation analyses, and moderation analyses were used to examine distributions of opportunity, associations between access and psychosocial variables, and predictors of intention to continue. The analyses were exploratory and associative rather than causal. Chi-square tests of independence were used to examine associations between categorical variables. Binary logistic regression was used to examine predictors of first-choice allocation. Predictors included gender, private lessons, home instrument access, prior junior high school music participation, socioeconomic status proxy, years of music experience, and ensemble type.

One-way analysis of variance was used to examine whether rehearsal climate variables differed by ensemble type: choir only, band only, and both choir and band. Independent-samples t-tests compared psychosocial outcomes across first-choice allocation status and leadership/visibility status. Pearson correlations examined associations amongst allocation satisfaction, inclusive discourse, leadership opportunities, belonging, self-efficacy, stereotype experiences, identity safety, and intention to continue. Multiple linear regression examined predictors of intention to continue. Mediation analyses were conducted using Hayes’ PROCESS macro, Model 4, with 5,000 bias-corrected bootstrap samples and 95% confidence intervals (Hayes, 2022). Three theoretically specified mediation models were tested: inclusive discourse → belonging → intention to continue; leadership opportunities → self-efficacy → intention to continue; and stereotype experiences → identity safety → intention to continue. Moderation analysis used PROCESS Model 1 to examine whether inclusive discourse moderated the association between first-choice allocation and belonging.

Analyses were conducted using available valid cases for each procedure. No imputation was applied. Results involving small gender categories were interpreted cautiously because small cell sizes can produce unstable estimates and increase disclosure risk. The analyses were exploratory and hypothesis-generating. No blanket Bonferroni correction was applied; interpretation emphasised effect sizes, theoretical coherence, and convergence across data sources.

### Qualitative analysis

Qualitative data from student focus groups and director interviews were analysed using thematic analysis informed by Braun and Clarke’s approach (Braun and Clarke, 2006, 2021). The analysis combined deductive attention to the gendered opportunity pathways framework with inductive openness to patterns emerging from participants’ accounts. Transcripts were read repeatedly for familiarisation. Initial coding focused on recurring patterns related to material constraints, unequal access, teacher-directed allocation, unclear criteria, perceived “fit” expectations, leadership and visibility, rehearsal correction and feedback, peer teasing and support, gendered assumptions, belonging, identity safety, self-efficacy, recognition, and continuation (Full outputs are provided in Supplementary Table S17).

Codes were reviewed and refined into themes that captured how students and directors explained, contextualised, or complicated the quantitative patterns. The final qualitative themes reported in the findings were material constraints and unequal access; teacher-directed allocation and unclear criteria; gendered “fit” assumptions; and peer support, teasing, and belonging. Quoted excerpts were selected because they illustrated recurrent patterns across focus groups and interviews or provided clarifying examples of how opportunity pathways were experienced in everyday ensemble practice. The qualitative analysis was used to explain and contextualise statistical patterns rather than to quantify prevalence.

### Mixed-methods integration

Mixed-methods integration occurred after separate quantitative and qualitative analyses. Integration was conducted through triangulation and a joint display that aligned findings from the survey, document audit, structured rehearsal observations, interviews, and focus groups. The integration examined convergence, complementarity, and expansion across data sources. The joint display produced integrated interpretations, or meta-inferences, about how material access, allocation practices, rehearsal interaction patterns, leadership opportunities, and psychosocial experiences formed linked gendered opportunity pathways.

### Researcher positionality and reflexivity

The researcher’s professional background in Ghanaian music education and prior work on school marching bands provided contextual familiarity with ensemble routines, terminology, authority structures, and school performance practices. This familiarity supported interpretation of the educational and cultural context of choir and marching band participation. At the same time, insider knowledge created potential risks of taken-for-granted assumptions about school authority, gender norms, rehearsal discipline, and music participation. To mitigate these risks, the study used multiple data sources, structured observation categories, anonymised transcript excerpts, and a mixed-methods joint display to compare evidence across survey, audit, observation, interview, and focus group data.

## Results

This section presents findings from the student survey, document audit, structured rehearsal observations, director interviews, and student focus groups. Results are organised around the study’s gendered opportunity pathways framework: sample characteristics and psychosocial descriptives; allocation, access, and first-choice attainment; leadership/visibility and recognition; rehearsal interaction patterns and climate; psychosocial predictors of continuation; and mixed-methods integration. Quantitative analyses are interpreted as exploratory and associative rather than causal.

### Sample characteristics and psychosocial descriptives

The sample comprised 236 students from seven senior high schools in the Central Region of Ghana. School representation was balanced, with each school contributing between 13.6 and 14.8% of the sample. Students were in Year 1, Year 2, and Year 3 and participated in choir, marching band, or both. Prior junior high school music participation was reported by 129 students, 54.7%, while 107 students, 45.3%, reported no prior junior high school music participation. The socioeconomic status proxy based on regular pocket money for school needs was distributed as follows: low pocket-money category, 71 students, 30.1%; middle pocket-money category, 109 students, 46.2%; and high pocket-money category, 56 students, 23.7%. Mean years of music experience was 3.42, with a standard deviation of 1.86 (see Table 1).

**Table 1:** Sample characteristics and participation profile, *N* = 236.

Variable	Category	*n*	%
School	SHS1–SHS7	32–35 per school	13.6–14.8
Year	Year 1	88	37.3
	Year 2	80	33.9
	Year 3	68	28.8
Gender identity	Girl	118	50.0
	Boy	110	46.6
	Non-binary/gender-diverse	5	2.1
	Prefer not to say	3	1.3
Ensemble participation	Choir only	113	47.9
	Band only	91	38.6
	Both choir and band	32	13.6
Private lessons	Yes	54	22.9
	No	182	77.1
Home instrument access	Yes	88	37.3
	No	148	62.7
Prior JHS music participation	Yes	129	54.7
	No	107	45.3
SES proxy	Low	71	30.1
	Middle	109	46.2
	High	56	23.7
Disability/learning need disclosed	Yes	14	5.9
	No/not disclosed	222	94.1
Years of music experience	Mean, SD	3.42	1.86

Percentages may not total exactly 100 because of rounding. JHS, junior high school. SES proxy was based on regular pocket money for school needs.

Psychosocial scales showed acceptable to good internal consistency. Mean scores were above the scale midpoint for belonging, music self-efficacy, identity safety, intention to continue, musical identity, and perceived recognition. Stereotype experiences were also slightly above the midpoint, indicating that stereotype-related comments, cues, or assumptions were reported by some students (see Table 2).

**Table 2:** Psychosocial descriptives and reliability, 1–5 composites, *N* = 236.

Scale	Items	Cronbach’s alpha	Mean	SD
Belonging	5	0.86	3.84	0.66
Music self-efficacy	4	0.82	3.71	0.62
Stereotype experiences	5	0.79	3.12	0.73
Identity safety	4	0.81	3.58	0.71
Intention to continue	3	0.84	3.90	0.77
Musical identity	4	0.83	3.76	0.68
Perceived recognition	4	0.80	3.49	0.72

### Allocation, access, and first-choice attainment

Overall, 146 students, 61.9%, reported receiving their first-choice instrument or role, while 90 students, 38.1%, did not. First-choice attainment varied descriptively by gender: 56.8% of girls, 69.1% of boys, 40.0% of non-binary/gender-diverse students, and 33.3% of students who preferred not to say reported receiving their first choice. The overall association between gender and first-choice allocation was not statistically significant, χ^2^ (3, *N* = 236) = 5.78, *p* = 0.123, *V* = 0.156. Because expected counts were small in the non-binary/gender-diverse and prefer-not-to-say categories, this full-category test should be interpreted cautiously. Amongst students who did not receive their first choice, the most frequently reported reason was that the preferred instrument or role was unavailable, 61.1%. Other reported reasons were cost or fees, 43.3%; teacher/director-directed placement, 36.7%; perceived “fit” expectations, including physicality, voice, or suitability, 26.7%; timetable constraints, 18.9%; and peer influence, 13.3%. Because students could select more than one reason, percentages exceed 100%.

Prior access to musical resources was associated with first-choice allocation. Students with private lessons were more likely to receive their first choice than those without private lessons, 75.9% versus 57.7%, χ^2^ (1, *N* = 236) = 5.87, *p* = 0.015, *ϕ* = 0.158. Students with home instrument access were also more likely to receive their first choice, 72.7% versus 55.4%, χ^2^ (1, *N* = 236) = 6.96, *p* = 0.008, *ϕ* = 0.172. Prior junior high school music participation was similarly associated with first-choice allocation: 68.2% of students with prior JHS music participation received their first choice, compared with 54.2% of those without prior JHS music participation, χ^2^ (1, *N* = 236) = 4.70, *p* = 0.030, *ϕ* = 0.141.

A binary logistic regression examined predictors of first-choice allocation while adjusting for selected access and background variables. Private lessons and years of music experience were statistically associated with higher odds of receiving one’s first-choice instrument or role. Students with private lessons had approximately twice the odds of receiving their first choice, *B* = 0.71, *p* = 0.037, *Exp*(*B*) = 2.03. Years of music experience were also significant, *B* = 0.19, *p* = 0.018, *Exp*(*B*) = 1.21. Home instrument access approached conventional significance, *B* = 0.58, *p* = 0.053, *Exp*(*B*) = 1.79. Gender, prior junior high school music participation, SES proxy, and ensemble type were not statistically significant in the adjusted model. Model fit statistics were -2*LL* = 296.42, Cox and Snell *R^2^* = 0.118, Nagelkerke *R^2^* = 0.160, and overall classification accuracy of 68.2%.

### Leadership/visibility and recognition

Leadership and visibility opportunities were unevenly distributed during the audited performance cycle. The document audit showed that 95 students, 40.3%, held at least one leadership/visibility role. Boys had the highest leadership/visibility rate, 50.0%, compared with 33.1% of girls, 20.0% of non-binary/gender-diverse students, and 0.0% of students who preferred not to say. The association between gender and leadership/visibility was statistically significant, χ^2^ (3, *N* = 236) = 9.76, *p* = 0.021, *V* = 0.203. Because two gender categories were very small, this finding should be interpreted cautiously and not overextended to gender-diverse students. Leadership/visibility varied descriptively by ensemble type: 34.5% of choir-only students, 46.2% of band-only students, and 43.8% of students participating in both choir and band held leadership/visibility roles. However, this association was not statistically significant, χ^2^ (2, *N* = 236) = 3.02, *p* = 0.221.

The document audit further showed that 53 students, 22.5%, held a solo/featured role during the audited performance cycle. Amongst girls, 16.1% held a solo/featured role, compared with 27.3% of boys. The Girl–Boy comparison indicated a statistically significant difference, χ^2^ (1, *N* = 228) = 4.13, *p* = 0.042. Small-cell values for non-binary/gender-diverse and prefer-not-to-say students are reported only in aggregate or suppressed where needed to protect confidentiality (see Table 3).

**Table 3:** Key opportunity distributions and logistic regression predicting first-choice allocation.

Indicator/Predictor	Key result	Test/model statistic
First-choice allocation	146/236, 61.9%	—
First-choice allocation by gender	Girls: 56.8%; Boys: 69.1%; Non-binary/GD: 40.0%; Prefer not to say: 33.3%	χ^2^ (3, *N* = 236) = 5.78, *p* = 0.123
Leadership/visibility role	95/236, 40.3%	—
Leadership/visibility by gender	Girls: 33.1%; Boys: 50.0%; Non-binary/GD: 20.0%; Prefer not to say: 0.0%	χ^2^ (3, *N* = 236) = 9.76, *p* = 0.021
Leadership/visibility by ensemble	Choir only: 34.5%; Band only: 46.2%; Both: 43.8%	χ^2^ (2, *N* = 236) = 3.02, p = 0.221
Solo/featured role	53/236, 22.5%	—
Solo/featured role, Girl–Boy comparison	Girls: 16.1%; Boys: 27.3%	χ^2^ (1, *N* = 228) = 4.13, *p* = 0.042
Private lessons predicting first-choice allocation	*B* = 0.71, *Exp*(*B*) = 2.03	*p* = 0.037
Home instrument predicting first-choice allocation	*B* = 0.58, *Exp* (*B*) = 1.79	p = 0.053
Years of music experience predicting first-choice allocation	*B* = 0.19, *Exp*(*B*) = 1.21	*p* = 0.018
Logistic regression model fit	Nagelkerke *R^2^* = 0.160; classification accuracy = 68.2%	−2*LL* = 296.42

Non-binary/GD, non-binary/gender-diverse. Small-cell gender findings are interpreted cautiously. Full outputs are provided in Supplementary Tables S2–S4 and S10.

Qualitative accounts contextualised these allocation and leadership patterns. A band director explained, “We have limited instruments. Sometimes I assign based on what is available, not what they want. If we had resources, the choices would be wider.” One student linked private access to faster progress: “If you have your own instrument or you pay for sticks, you progress fast. Some of us wait to share, so you cannot practice enough.” Another student described a gendered “fit” assumption in allocation: “I wanted Tuba, but they said it is heavy and I should take tenor drum. Later, I saw a boy in my class, but he was allowed to try the Tuba.” Leadership was also described as tied to noticeability and public confidence: “If you talk loud and you are confident, the master notices you.”

### Rehearsal interaction patterns and rehearsal climate

Structured observations were conducted across 14 rehearsals, comprising seven choir rehearsals and seven marching band rehearsals. Inter-rater reliability estimates indicated acceptable to strong agreement across observation categories. Cohen’s kappa values ranged from *κ* = 0.65 for peer teasing incidents to *κ* = 0.88 for feedback recipient gender classification. Intraclass correlations for event counts ranged from *ICC* = 0.69 to *ICC* = 0.87, indicating moderate to good consistency between observers. Observation counts suggested that marching band rehearsals were more correction-heavy and command-oriented than choir rehearsals. Band rehearsals had more technical corrective feedback, behaviour/discipline correction, interruptions or over-talk, leadership invitations, and peer teasing. Choir rehearsals had more specific teacher praise and student-initiated musical suggestions.

Survey-based rehearsal climate measures showed a similar pattern. One-way ANOVAs indicated statistically significant differences by ensemble type for peer encouragement, correction/criticism, inclusive discourse, and stereotype comments frequency. Teacher feedback quality did not differ significantly by ensemble type (see Table 4).

**Table 4:** Rehearsal observation counts/rates and climate ANOVA summary.

Observed interaction metric	Choir count	Choir rate/Rehearsal	Band count	Band rate/Rehearsal
Technical corrective feedback	98	14.0	121	17.3
Behaviour/discipline correction	39	5.6	102	14.6
Specific teacher praise	47	6.7	38	5.4
Talent/innate-ability statements	16	2.3	23	3.3
Student-initiated musical suggestions	31	4.4	19	2.7
Interruptions/over-talk incidents	24	3.4	38	5.4
Leadership invitations	22	3.1	36	5.1
Peer teasing incidents	13	1.9	23	3.3

Survey rehearsal climate outcome	Direction of mean pattern	ANOVA result	Effect size
Teacher feedback quality	No significant ensemble difference	*F* (2, 233) = 1.64, *p* = 0.196	*ηp^2^* = 0.014
Peer encouragement	Higher in choir-only than band-only	*F* (2, 233) = 4.26, p = 0.015	*ηp^2^* = 0.035
Correction/criticism	Higher in band-only than choir-only	*F* (2, 233) = 8.72, p < 0.001	*ηp^2^* = 0.070
Inclusive discourse	Higher in choir-only than band-only	*F* (2, 233) = 4.91, *p* = 0.008	*ηp^2^* = 0.040
Stereotype comments frequency	Higher in band-only than choir-only	*F* (2, 233) = 5.08, p = 0.007	*ηp^2^* = 0.042

Observation rates were calculated using seven rehearsals per ensemble type. Detailed observation counts and rehearsal climate descriptives are provided in Supplementary Tables S5 and S6.

Feedback visibility also differed descriptively by ensemble type. In band rehearsals, 60% of feedback events were directed to boys and 38% to girls, with 2% coded as unclear/mixed. In choir rehearsals, 52% of feedback events were directed to boys and 46% to girls, with 2% coded as unclear/mixed. These observation data indicate visible instructional attention during the observed rehearsals and should not be interpreted as a comprehensive measure of feedback across the school year. Qualitative accounts clarified how students experienced rehearsal interaction patterns. One student who participated in both ensembles stated, “Band rehearsal is strict. Sometimes it helps, but sometimes the shouting makes you afraid to make mistakes.” Another student linked peer climate to continuation: “When seniors support you, you stay. When they tease you, you stop. Peer behaviour is the biggest thing.” A girl in band described correction as gendered in meaning: “When I make a mistake, they correct me like ‘you see why we do not put girls there.’ But when boys make mistake they only say ‘practice more.’”

### Psychosocial outcomes and intention to continue

Students who received their first-choice instrument or role reported significantly more positive psychosocial outcomes than students who did not. Independent-samples t-tests showed significant differences for allocation satisfaction, belonging, self-efficacy, identity safety, and intention to continue. The largest difference was for allocation satisfaction, with students who received their first choice reporting higher satisfaction, *M* = 4.02, *SD* = 0.82, than students who did not, *M* = 3.12, *SD* = 0.89, *t*(234) = −7.18, *p* < 0.001, *d* = 0.96. First-choice allocation was also associated with higher belonging, *t*(234) = −3.96, *p* < 0.001, *d* = 0.53; self-efficacy, *t*(234) = −2.78, *p* = 0.006, *d* = 0.37; identity safety, *t*(234) = −2.74, *p* = 0.007, *d* = 0.37; and intention to continue, *t*(234) = −3.59, *p* < 0.001, *d* = 0.48.

Leadership/visibility status was similarly associated with psychosocial outcomes. Students with leadership/visibility roles reported significantly higher belonging, *t*(234) = −3.86, *p* < 0.001, *d* = 0.51; self-efficacy, *t*(234) = −6.12, *p* < 0.001, *d* = 0.81; musical identity, *t*(234) = −5.22, *p* < 0.001, *d* = 0.69; perceived recognition, *t*(234) = −6.41, *p* < 0.001, *d* = 0.85; and intention to continue, *t*(234) = −4.20, *p* < 0.001, *d* = 0.56, than students without leadership/visibility roles. The largest difference was in perceived recognition, with students holding leadership roles reporting higher recognition, *M* = 3.84, *SD* = 0.62, than those without such roles, *M* = 3.25, *SD* = 0.70.

Pearson correlations showed theoretically coherent associations Amongst allocation, rehearsal climate, leadership opportunities, psychosocial experiences, and continuation intention. Belonging was strongly associated with intention to continue, *r* = 0.58, *p* < 0.01, as was self-efficacy, *r* = 0.52, *p* < 0.01. Inclusive discourse was positively associated with belonging, *r* = 0.51, *p* < 0.01, and identity safety, *r* = 0.53, *p* < 0.01. Stereotype experiences were negatively associated with inclusive discourse, *r* = −0.42, *p* < 0.01; identity safety, *r* = −0.50, *p* < 0.01; belonging, *r* = −0.39, *p* < 0.01; and intention to continue, *r* = −0.34, *p* < 0.01. Leadership opportunities were positively associated with self-efficacy, *r* = 0.49, *p* < 0.01, and intention to continue, *r* = 0.38, *p* < 0.01. The full correlation matrix is provided in Supplementary Table S8.

A multiple linear regression model examined predictors of intention to continue. The model explained approximately 49% of the variance in continuation intention, *R* = 0.70, *R^2^* = 0.49, adjusted *R^2^* = 0.47. Significant predictors were belonging, *β* = 0.29, *p* < 0.001; self-efficacy, *β* = 0.25, *p* < 0.001; allocation satisfaction, *β* = 0.17, *p* = 0.004; identity safety, *β* = 0.16, *p* = 0.011; and leadership opportunity count, *β* = 0.11, *p* = 0.043. Stereotype experiences were negatively related to intention to continue but were not statistically significant in the adjusted model, *β* = −0.08, *p* = 0.138. Gender, private lessons, and home instrument access were also not significant predictors in the adjusted model.

Mediation analyses were conducted using Hayes’ PROCESS Model 4 with 5,000 bias-corrected bootstrap samples and 95% confidence intervals. These analyses are interpreted as cross-sectional indirect associations rather than causal mediation. Belonging mediated the association between inclusive discourse and intention to continue, indirect effect = 0.22, 95% CI [0.15, 0.31]. Self-efficacy mediated the association between leadership opportunity count and intention to continue, indirect effect = 0.06, 95% CI [0.02, 0.10]. Identity safety mediated the association between stereotype experiences and intention to continue, indirect effect = −0.19, 95% CI [−0.28, −0.12].

A moderation analysis using Hayes’ PROCESS Model 1 examined whether inclusive discourse moderated the association between first-choice allocation and belonging. The interaction was statistically significant, *B* = −0.16, *p* = 0.023, indicating that the belonging gap between students who did and did not receive their first-choice instrument or role was smaller when inclusive discourse was higher (see Table 5).

**Table 5:** Regression, mediation, and moderation summary.

Analysis	Key predictors/Pathways	Result	Interpretation
Multiple regression predicting intention to continue	Belonging	*β* = 0.29, *p* < 0.001	Higher belonging was associated with stronger intention to continue.
	Self-efficacy	*β* = 0.25, *p* < 0.001	Higher self-efficacy was associated with stronger intention to continue.
	Allocation satisfaction	*β* = 0.17, *p* = 0.004	Greater allocation satisfaction was associated with stronger continuation intention.
	Identity safety	*β* = 0.16, *p* = 0.011	Higher identity safety was associated with stronger continuation intention.
	Leadership opportunity count	*β* = 0.11, *p* = 0.043	More leadership opportunities were associated with stronger continuation intention.
	Model fit	*R^2^* = 0.49; adjusted *R^2^* = 0.47	Model explained approximately 49% of variance.
Mediation model 1	Inclusive discourse → Belonging → Intention to continue	Indirect effect = 0.22, 95% CI [0.15, 0.31]	Belonging partially mediated this association.
Mediation model 2	Leadership opportunities → Self-efficacy → Intention to continue	Indirect effect = 0.06, 95% CI [0.02, 0.10]	Self-efficacy partially mediated this association.
Mediation model 3	Stereotype experiences → Identity safety → Intention to continue	Indirect effect = −0.19, 95% CI [−0.28, −0.12]	Identity safety mediated the negative association.
Moderation model	First-choice allocation × Inclusive discourse predicting belonging	*B* = −0.16, *p* = 0.023	Inclusive discourse reduced the belonging gap linked to not receiving first choice.

Mediation and moderation analyses used Hayes’ PROCESS macro with 5,000 bias-corrected bootstrap samples and 95% confidence intervals. Results are cross-sectional and associative.

### Mixed-methods integration

Mixed-methods integration was conducted through triangulation and joint display analysis. Quantitative, observational, and qualitative findings converged around four major themes: material constraints and unequal access; teacher-directed allocation and unclear criteria; gendered “fit” assumptions; and peer support, teasing, and belonging (see Table 6).

**Table 6:** Mixed-methods joint display.

Quantitative/Observational finding	Qualitative evidence	Integrated interpretation
Private lessons, home instrument access, prior JHS music participation, and years of experience were associated with first-choice allocation.	Students and directors described instrument scarcity and the advantage of owning instruments or paying for materials.	Material access shaped allocation pathways by influencing practice opportunity, preparedness, and visibility.
Boys were more likely than girls to hold leadership/visibility roles.	Students linked leadership to loudness, confidence, discipline, and public authority.	Leadership/visibility appeared to function as a recognition process shaped by competence, noticeability, and gendered expectations.
Solo/featured roles were less common and more frequent among boys than girls.	Students described solo allocation as opaque and front positioning as a visibility bottleneck.	Public visibility was experienced as a high-status opportunity shaped by selection transparency and positioning.
Band rehearsals had more behaviour correction, peer teasing, leadership invitations, and boy-directed feedback.	Students described band as strict, command-oriented, and sometimes intimidating.	Band settings offered visible leadership opportunities while also increasing correction, pressure, and peer surveillance.
Stereotype experiences were negatively associated with identity safety, belonging, and intention to continue.	Students described gendered comments around strength, suitability, and mistakes.	Stereotype-related interaction may reduce identity safety by making ordinary mistakes feel identity-diagnostic.
Belonging and self-efficacy were strong predictors of intention to continue.	Students described staying when peers, seniors, and teachers were supportive.	Continuation was linked not only to musical skill but also to recognition, support, and psychosocial safety.
Inclusive discourse moderated the association between first-choice allocation and belonging.	Some students reported continuing despite disappointment when teachers or peers remained supportive.	Inclusive rehearsal climates appeared to reduce the belonging gap associated with not receiving one’s preferred role.

Overall, the integrated evidence suggests that students’ ensemble pathways were shaped by linked material, institutional, interactional, and psychosocial processes. Access to instruments and prior musical resources was associated with first-choice allocation; leadership/visibility and solo/featured roles were unevenly distributed; band rehearsals involved more correction and teasing; and belonging, self-efficacy, identity safety, allocation satisfaction, and recognition were associated with students’ intention to continue. Qualitative accounts clarified how these patterns were experienced in everyday rehearsal life through scarcity, unclear allocation criteria, gendered “fit” assumptions, peer teasing, and support.

## Discussion

This study examined gendered opportunity pathways in Ghanaian senior high school choir and marching band ensembles using a convergent mixed-methods design. The central contribution is that ensemble participation was not shaped by a single allocation decision or isolated act of inclusion/exclusion. Rather, students’ pathways were formed through linked material, institutional, interactional, and psychosocial processes: access to instruments and prior training, first-choice allocation, leadership/visibility roles, solo/featured opportunities, rehearsal interaction patterns, peer climate, recognition, belonging, self-efficacy, identity safety, and intention to continue.

Several findings are central. First, first-choice allocation was incomplete: 61.9% of students received their preferred instrument or role, while 38.1% did not. Gender differences in first-choice allocation were descriptive but not statistically significant in the full-category test. Second, access-related variables were important. Private lessons and years of music experience were associated with higher odds of receiving first-choice allocation in the logistic regression model, while home instrument access approached conventional significance. Third, leadership/visibility roles and solo/featured roles were unevenly distributed, with boys more likely than girls to hold these visible forms of recognition. Fourth, choir and marching band rehearsals showed different interactional climates: band rehearsals had higher levels of correction/criticism, peer teasing, leadership invitations, and boy-directed feedback, while choir rehearsals showed more specific praise and student-initiated suggestions. Fifth, psychosocial variables mattered strongly. Belonging, self-efficacy, allocation satisfaction, identity safety, and leadership opportunity count predicted intention to continue, with the model explaining approximately 49% of variance.

The mediation and moderation analyses further clarified the study’s pathway-oriented interpretation. Belonging mediated the association between inclusive discourse and intention to continue; self-efficacy mediated the association between leadership opportunities and intention to continue; and identity safety mediated the association between stereotype experiences and intention to continue. Inclusive discourse also moderated the association between first-choice allocation and belonging, suggesting that the belonging gap linked to not receiving one’s preferred role was smaller when rehearsal discourse was more inclusive. Because the study was cross-sectional, these findings should be interpreted as theoretically informed indirect and buffering associations, not as evidence of causal mediation or temporal ordering.

### Gendered opportunity pathways in allocation and leadership

The first-choice allocation findings require careful interpretation. Boys reported higher first-choice attainment than girls, and students in the small non-binary/gender-diverse and prefer-not-to-say categories reported lower attainment descriptively. However, the full-category chi-square test was not statistically significant. This means the data do not support a strong claim that gender independently predicted first-choice allocation at the bivariate level. The descriptive pattern nevertheless raises an equity-relevant question about how preference, perceived suitability, and institutional decision-making intersect in constrained ensemble settings. The clearer gendered pattern emerged in leadership/visibility. Leadership/visibility roles were significantly associated with gender, and boys were more likely than girls to hold such roles. Boys were also more likely than girls to hold solo/featured roles in the Girl–Boy comparison.

These findings align with prior music education research showing that instruments, voices, musical roles, and authority positions often carry gendered meanings (Abeles and Porter, 1978; Green, 1997; Hallam et al., 2008; Sergeant and Himonides, 2014). The qualitative evidence helps explain why leadership should not be interpreted simply as a neutral reward for ability. Students described leadership as linked to loudness, confidence, discipline, visibility, and public authority. Such accounts are consistent with the idea that schools communicate recognition through everyday routines, expectations, and informal norms, not only through official policies. At the same time, leadership may also reflect seniority, attendance, technical proficiency, confidence, director judgement, role availability, and performance-cycle demands. The findings therefore suggest a gendered pattern in leadership/visibility, but they do not establish gender as the sole or causal explanation.

## Material access, prior experience, and first-choice allocation

The allocation findings also show that access to musical resources matters. In bivariate analyses, private lessons, access to a home instrument, and prior junior high school music participation were each associated with first-choice allocation. In the logistic regression model, private lessons and years of music experience remained statistically significant predictors, while home instrument access approached conventional significance. Prior junior high school music participation was significant in bivariate analysis but not in the adjusted model, suggesting that its association with allocation may overlap with other access-related factors such as accumulated experience, private preparation, or home resources. These findings point to a cumulative advantage process in school music participation. Students who have private lessons, more years of experience, or access to a home instrument may enter senior high school ensembles with greater technical readiness, confidence, and familiarity with rehearsal expectations. Such advantages can shape allocation outcomes without implying that directors intentionally privilege already-resourced students. Rather, unequal access can become embedded in apparently practical decisions about readiness, reliability, and role suitability.

This interpretation is consistent with broader accounts of cultural capital and unequal educational opportunity. Prior access to valued skills, materials, and family-supported learning opportunities can influence how students are recognised within school systems (Bourdieu, 1986; Lareau, 2011). In music ensembles, these advantages may appear through access to instruments, private tuition, practice time, repair support, and the ability to demonstrate competence before formal allocation decisions are made.

## Rehearsal interaction patterns, identity safety, and belonging

The observation and ANOVA findings showed that choir and marching band rehearsals functioned as distinct interactional environments. Band rehearsals had higher counts of technical correction, behaviour/discipline correction, interruptions or over-talk, leadership invitations, peer teasing, and boy-directed feedback. Choir rehearsals showed more specific teacher praise and student-initiated musical suggestions. Survey measures converged with these observation patterns: band-only students reported higher correction/criticism and more frequent stereotype comments, whereas choir-only students reported higher peer encouragement and inclusive discourse.

These findings require nuance. Correction is not inherently harmful in music education. Technical correction is central to musical development, ensemble precision, and performance quality. Marching bands may also require more behavioural coordination because they involve movement, alignment, timing, command structures, safety, and public display. However, correction becomes equity-relevant when it is unevenly distributed, delivered in ways that heighten fear of mistakes, or interpreted through gendered assumptions.

Stereotype experiences were negatively correlated with identity safety, belonging, inclusive discourse, and intention to continue. Identity safety mediated the association between stereotype experiences and intention to continue. This suggests that stereotype-related comments or cues may matter because they shape whether students feel safe being visible, corrected, and evaluated.

This interpretation aligns with stereotype-threat and identity-safety research showing that identity-relevant cues can affect how students interpret evaluative settings (Steele, 1997; Spencer et al., 1999; Davies et al., 2002; Steele et al., 2002). It also resonates with work showing that institutional and interpersonal cues can signal whether students are likely to be respected, judged fairly, or exposed to identity-based devaluation (Davies et al., 2005; Purdie-Vaughns et al., 2008). In this sense, belonging is not only an individual feeling but also an interpretation of repeated social and institutional signals within the learning environment (Walton and Brady, 2017).

## Psychosocial mechanisms of continuation

The psychosocial findings show that continuation intention was strongly linked to students’ sense of belonging, competence, recognition, and safety. Students who received their first-choice instrument or role reported higher allocation satisfaction, belonging, self-efficacy, identity safety, and intention to continue. Students with leadership/visibility roles reported higher belonging, self-efficacy, musical identity, perceived recognition, and intention to continue.

The multiple regression model strengthened this interpretation. Belonging and self-efficacy were the strongest predictors of intention to continue, with allocation satisfaction, identity safety, and leadership opportunity count following. This pattern aligns with educational psychology research indicating that students are more likely to persist when they feel they belong, believe they can succeed, and perceive their learning environment as supportive and meaningful (Bandura, 1997; Deci and Ryan, 2000; Goodenow, 1993a, 1993b; Eccles and Roeser, 2011; Walton and Cohen, 2007). It is also consistent with music education research showing that self-efficacy, performance confidence, musical identity, and supportive participation contexts are important for students’ musical engagement and persistence (McPherson and McCormick, 2006; Hallam et al., 2012).

Because these mediation and moderation analyses are based on cross-sectional data, they cannot establish temporal or causal mechanisms. Nevertheless, they provide theoretically coherent evidence that belonging, self-efficacy, and identity safety are plausible psychosocial pathways connecting rehearsal climate, recognition, stereotype experiences, and continuation intention.

## Mixed-methods Meta-inference

The mixed-methods meta-inference is that gendered opportunity pathways were distributed across multiple moments of ensemble life rather than located in a single decision point. The survey and regression analyses identified distributional patterns: access variables were associated with first-choice allocation, leadership/visibility roles were unevenly distributed, and psychosocial predictors were associated with intention to continue. The observation data showed how everyday rehearsal interaction patterns differed by ensemble type, particularly in correction, teasing, leadership invitations, and feedback visibility. The qualitative accounts explained how students and directors interpreted these patterns through scarcity, teacher-directed allocation, perceived “fit,” peer support, teasing, confidence, discipline, and public authority.

This pathway account contributes to music education research by extending gender analysis beyond instrument choice alone. Equity in ensemble participation requires attention not only to who joins, but also to who is placed, heard, corrected, praised, invited to lead, protected from ridicule, and publicly recognised.

## Implications for music education practice and policy

The findings point to several implications for senior high school ensemble directors, music educators, school leaders, and policy actors.

First, allocation systems should be made more transparent. In resource-constrained ensembles, not every student can receive a preferred instrument or role immediately. However, schools can reduce perceived unfairness by explaining allocation criteria, allowing trial periods, offering reassessment opportunities, and communicating pathways for future mobility. Where physicality, voice, range, endurance, or technical suitability are used as criteria, they should be applied consistently and developmentally rather than as fixed assumptions about gender or body type.

Second, leadership/visibility roles should be treated as developmental opportunities, not only as rewards for students who are already confident or visible. Schools can monitor who receives section leadership, solo/featured roles, front positioning, student conducting, demonstration tasks, and command roles. Rotating low-stakes leadership opportunities may help broaden access to recognition and support self-efficacy.

Third, resource provision should be treated as an equity issue. Private lessons, years of experience, and home instrument access were associated with first-choice allocation, suggesting that unequal preparatory resources may shape later opportunity. Schools can respond by providing shared instruments, repair funds, practice schedules, supervised access to instruments, sticks and materials, and support for students who cannot afford private resources.

Fourth, rehearsal climate should be understood as part of inclusive pedagogy. Directors should use feedback that is specific, constructive, and musically focused. Correction should avoid linking mistakes to gendered assumptions about strength, suitability, sound, confidence, or authority. Peer teasing should be addressed directly through rehearsal norms, senior-student mentoring, and consistent intervention.

Finally, belonging, self-efficacy, identity safety, and perceived recognition should be treated as core ensemble outcomes. Music education often emphasises performance quality, discipline, and public representation. These remain important. However, the present findings show that students’ intention to continue is also linked to whether they feel recognised, capable, safe, and included.

## Limitations and future research

Several limitations should guide interpretation. First, the study used a cross-sectional convergent mixed-methods design. The regression, mediation, and moderation analyses identify associations and theoretically informed pathways, but they do not establish causal direction or temporal ordering. The mediation models should therefore be understood as cross-sectional indirect associations, not proof that inclusive discourse, leadership opportunities, or stereotype experiences caused later continuation outcomes.

Second, the study was conducted in seven senior high schools in one region of Ghana. Although the schools were selected because they maintained both choir and marching band ensembles, the findings should not be generalised to all Ghanaian schools. Criterion-based sampling may have overrepresented schools with stronger or more visible music traditions.

Third, the non-binary/gender-diverse and prefer-not-to-say groups were small. These students were retained descriptively, but subgroup inference was limited to avoid unstable estimates and re-identification risk.

Fourth, structured observations captured 14 rehearsals, one choir and one marching band rehearsal per school. These observations provide useful indicators of rehearsal interaction patterns, but they cannot represent all rehearsals across a full academic year, performance cycle, examination period, or director style. Some categories, including peer teasing, interruptions, and talent/innate-ability statements, involved interpretive judgement despite acceptable reliability estimates.

Fifth, feedback visibility was coded by perceived gender during observation and was not equivalent to self-identified gender. These data should therefore be interpreted as descriptive indicators of visible instructional attention, not as definitive measures of gendered feedback distribution.

Sixth, several psychosocial constructs were measured using the same self-report questionnaire at one time point, creating possible common method variance. The mixed-methods design helped contextualise these associations, but it does not eliminate same-source bias.

Seventh, the quantitative analyses were exploratory and involved multiple statistical tests. No blanket multiple-comparison adjustment was applied. Findings should therefore be interpreted in relation to effect sizes, theoretical coherence, and convergence across data sources.

Eighth, the psychosocial scales were developed specifically for this study. Although they showed acceptable internal consistency and preliminary construct-validity evidence through factor analysis and theoretically coherent correlations, further validation is needed in larger and more diverse samples, including measurement invariance testing across gender, ensemble type, school context, and region.

Ninth, the leadership/visibility audit covered one performance cycle only. Leadership, solo/featured roles, and front positioning may vary across events, terms, repertoire, and school priorities. Finally, variables such as SES proxy and years of music experience may not fully capture the structural inequalities shaping students’ musical preparation, family support, transport, time, practice space, or access to private tuition.

Future research should examine gendered opportunity pathways longitudinally. Following students across terms or school years would allow researchers to investigate whether early allocation, practice access, feedback experiences, peer climate, and leadership invitations predict later self-efficacy, belonging, identity safety, visibility, and continuation or dropout.

Intervention studies are also needed. Researchers could examine whether transparent placement criteria, rotating leadership opportunities, anti-teasing protocols, peer mentoring, structured practice access, or director training in inclusive feedback improve belonging, identity safety, self-efficacy, and continuation intention.

Larger samples are needed to examine gender-diverse students’ experiences ethically and safely. Such studies should be designed with strong confidentiality protections, careful small-cell reporting, and attention to local sociocultural contexts. Future work should also use intersectional approaches that consider gender alongside socioeconomic status, private lessons, home instrument access, disability or learning needs, boarding/day status, school type, prior music participation, and years of experience.

## Conclusion

This study shows that Ghanaian senior high school music ensembles are not only performance spaces; they are social-psychological environments in which students encounter opportunity, recognition, evaluation, and belonging. Across the sampled schools, students’ pathways were shaped through access to instruments and prior preparation, first-choice allocation, leadership/visibility roles, solo/featured opportunities, rehearsal interaction patterns, peer climate, inclusive discourse, identity safety, self-efficacy, and perceived recognition.

The findings suggest that equity in school music ensembles depends on more than increasing participation. It requires attention to who receives preferred roles, who is heard, who is corrected constructively, who is praised, who is invited to lead, who is protected from teasing, and who is publicly recognised as musically capable. Even when material constraints prevent all students from receiving preferred instruments or roles, inclusive rehearsal climates may help protect belonging and support continuation. Building equitable ensemble environments, therefore, requires both resource attention and everyday pedagogical care: transparent allocation, fair pathways to visibility, developmental feedback, peer support, and rehearsal cultures in which all students can participate without identity-based scrutiny.

## Data Availability

The original contributions presented in the study are included in the article/Supplementary material, further inquiries can be directed to the corresponding author.
