# Supplementary material for: Who is placed, heard, and seen? Gendered opportunity pathways in Ghanaian senior high school choirs and marching bands
Source: Front Psychol. 2026 Aug 12;17:1913572. doi: 10.3389/fpsyg.2026.1913572 (PMC13506489; doi:10.3389/fpsyg.2026.1913572)
Supplement: Supplementary file 1 [file Supplementary_File_1.docx]

**Supplementary Material 1: Additional Quantitative Tables**

This supplementary file provides detailed descriptive and inferential outputs supporting the main manuscript. The tables include full demographic frequencies, crosstabulations, chi-square tests, access-variable comparisons, structured rehearsal observation counts and rates, rehearsal climate ANOVA outputs, independent-samples t-tests, correlations, PROCESS mediation and moderation outputs, logistic regression, and multiple regression results. Quantitative analyses were conducted in IBM SPSS Statistics. Mediation and moderation analyses were conducted using Hayes’ PROCESS macro. Simple mediation models used PROCESS Model 4, and the moderation model used PROCESS Model 1. PROCESS analyses used 5,000 bias-corrected bootstrap samples and 95% confidence intervals. Analyses are interpreted as exploratory and associative rather than causal.

**Supplementary Table S1: Full demographic frequency table**

Supplementary Table S1 presents the full demographic and participation profile of the student survey sample. The analytic sample comprised 236 students from seven senior high schools.

| **Variable** | **Category** | **n** | **%** |
| --- | --- | --- | --- |
| School | SHS1 | 34 | 14.4 |
|  | SHS2 | 33 | 14.0 |
|  | SHS3 | 32 | 13.6 |
|  | SHS4 | 35 | 14.8 |
|  | SHS5 | 34 | 14.4 |
|  | SHS6 | 33 | 14.0 |
|  | SHS7 | 35 | 14.8 |
| Year group | Year 1 | 88 | 37.3 |
|  | Year 2 | 80 | 33.9 |
|  | Year 3 | 68 | 28.8 |
| Gender identity | Girl | 118 | 50.0 |
|  | Boy | 110 | 46.6 |
|  | Non-binary/gender-diverse | 5 | 2.1 |
|  | Prefer not to say | 3 | 1.3 |
| Ensemble participation | Choir only | 113 | 47.9 |
|  | Band only | 91 | 38.6 |
|  | Both choir and band | 32 | 13.6 |
| Private lessons | No | 182 | 77.1 |
|  | Yes | 54 | 22.9 |
| Home instrument access | No | 148 | 62.7 |
|  | Yes | 88 | 37.3 |
| Prior JHS music participation | No | 107 | 45.3 |
|  | Yes | 129 | 54.7 |
| Disability/learning need disclosed | No/not disclosed | 222 | 94.1 |
|  | Yes | 14 | 5.9 |
| Socioeconomic status proxy | Low pocket-money category | 71 | 30.1 |
|  | Middle pocket-money category | 109 | 46.2 |
|  | High pocket-money category | 56 | 23.7 |
| Years of music experience | Mean, SD | 3.42 | 1.86 |

*Note. Percentages may not sum to exactly 100 because of rounding. JHS = junior high school. The socioeconomic status proxy was measured using students’ reported regularity of pocket money for school needs. Years of music experience is reported as mean and standard deviation.*

**Supplementary Table S2**

Gender identity vs first-choice allocation crosstab and chi-square output

Supplementary Table S2 presents the crosstabulation of gender identity by first-choice instrument/role allocation. Small gender categories are retained descriptively but interpreted cautiously because of small cell sizes.

| **Gender identity** | **First choice met: Yes** | **First choice met: No** | **Total** | **Row % Yes** |
| --- | --- | --- | --- | --- |
| Girl | 67 | 51 | 118 | 56.8 |
| Boy | 76 | 34 | 110 | 69.1 |
| Non-binary/gender-diverse | 2 | 3 | 5 | 40.0 |
| Prefer not to say | 1 | 2 | 3 | 33.3 |
| **Total** | **146** | **90** | **236** | **61.9** |

#### Chi-Square Tests

| **Test** | **Value** | **df** | **N** | **p** |
| --- | --- | --- | --- | --- |
| Pearson chi-square | 5.78 | 3 | 236 | .123 |
| Cramér’s V | .156 | — | 236 | .123 |

*Note. The Pearson chi-square includes all four gender categories. Because expected counts are small in the non-binary/gender-diverse and prefer-not-to-say categories, results involving these groups should be interpreted cautiously. Cramér’s V was calculated for the two-column crosstab.*

**Supplementary Table S3: Gender identity vs leadership/visibility role crosstab and chi-square output**

Supplementary Table S3 presents the crosstabulation of gender identity by leadership/visibility role status during the audited performance cycle.

| **Gender identity** | **Leadership/visibility: Yes** | **Leadership/visibility: No** | **Total** | **Row % Yes** |
| --- | --- | --- | --- | --- |
| Girl | 39 | 79 | 118 | 33.1 |
| Boy | 55 | 55 | 110 | 50.0 |
| Non-binary/gender-diverse | 1 | 4 | 5 | 20.0 |
| Prefer not to say | 0 | 3 | 3 | 0.0 |
| **Total** | **95** | **141** | **236** | **40.3** |

#### **Chi-Square Tests**

| **Test** | **Value** | **df** | **N** | **p** |
| --- | --- | --- | --- | --- |
| Pearson chi-square | 9.76 | 3 | 236 | .021 |
| Cramér’s V | .203 | — | 236 | .021 |

*Note. Leadership/visibility roles included section leader or assistant section leader, first-chair or lead position, drum major or band commander, student conductor, public rehearsal leader, named soloist or featured performer, or other documented visible responsibility. Small-cell gender categories are retained descriptively and interpreted cautiously. Cramér’s V was calculated for the two-column crosstab.*

**Supplementary Table S4: Access-variable crosstabs and chi-square tests predicting first-choice allocation**

Supplementary Table S4 presents crosstabulations and chi-square tests for access-related variables and first-choice allocation.

Supplementary Table S4a

Private lessons vs first-choice allocation

| **Private lessons** | **First choice met: Yes** | **First choice met: No** | **Total** | **Row % Yes** |
| --- | --- | --- | --- | --- |
| No | 105 | 77 | 182 | 57.7 |
| Yes | 41 | 13 | 54 | 75.9 |
| Total | 146 | 90 | 236 | 61.9 |

| **Test** | **Value** | **df** | **N** | **p** | **Phi / Cramér’s V** |
| --- | --- | --- | --- | --- | --- |
| Pearson chi-square | 5.87 | 1 | 236 | .015 | .158 |

**Supplementary Table S4b: Home instrument access × first-choice allocation**

| **Home instrument access** | **First choice met: Yes** | **First choice met: No** | **Total** | **Row % Yes** |
| --- | --- | --- | --- | --- |
| No | 82 | 66 | 148 | 55.4 |
| Yes | 64 | 24 | 88 | 72.7 |
| **Total** | **146** | **90** | **236** | **61.9** |

| **Test** | **Value** | **df** | **N** | **p** | **Phi / Cramér’s V** |
| --- | --- | --- | --- | --- | --- |
| Pearson chi-square | 6.96 | 1 | 236 | .008 | .172 |

**Supplementary Table S4c: Prior JHS music participation vs first-choice allocation**

| **Prior JHS music participation** | **First choice met: Yes** | **First choice met: No** | **Total** | **Row % Yes** |
| --- | --- | --- | --- | --- |
| No | 58 | 49 | 107 | 54.2 |
| Yes | 88 | 41 | 129 | 68.2 |
| **Total** | **146** | **90** | **236** | **61.9** |

| **Test** | **Value** | **df** | **N** | **p** | **Phi / Cramér’s V** |
| --- | --- | --- | --- | --- | --- |
| Pearson chi-square | 4.70 | 1 | 236 | .030 | .141 |

*Note. JHS = junior high school. Phi/Cramér’s V values were calculated.*

**Supplementary Table S5: Full structured rehearsal observation count table**

Supplementary Table S5 presents the full structured observation counts and rates per rehearsal by ensemble type. Fourteen rehearsals were observed: seven choir rehearsals and seven marching band rehearsals. Rates were calculated by dividing counts by seven for each ensemble type.

| **Observed interaction metric** | **Choir count** | **Choir rate/rehearsal** | **Band count** | **Band rate/rehearsal** | **Total count** |
| --- | --- | --- | --- | --- | --- |
| Teacher corrective feedback: technical | 98 | 14.0 | 121 | 17.3 | 219 |
| Teacher corrective feedback: behaviour/discipline | 39 | 5.6 | 102 | 14.6 | 141 |
| Specific teacher praise | 47 | 6.7 | 38 | 5.4 | 85 |
| “Talent” or innate-ability statements | 16 | 2.3 | 23 | 3.3 | 39 |
| Student-initiated musical suggestions | 31 | 4.4 | 19 | 2.7 | 50 |
| Interruptions or over-talk incidents | 24 | 3.4 | 38 | 5.4 | 62 |
| Leadership invitations | 22 | 3.1 | 36 | 5.1 | 58 |
| Audible peer teasing incidents | 13 | 1.9 | 23 | 3.3 | 36 |

### Feedback visibility by perceived gender

| **Ensemble type** | **Feedback directed to girls** | **Feedback directed to boys** | **Unclear/mixed** |
| --- | --- | --- | --- |
| Choir | 46.0% | 52.0% | 2.0% |
| Band | 38.0% | 60.0% | 2.0% |

*Note. Feedback visibility was coded by perceived gender during observation and was not treated as equivalent to students’ self-identified gender. Observation results are descriptive and reflect the sampled rehearsals only.*

**Supplementary Table S6: Rehearsal climate descriptive statistics and ANOVA results by ensemble type**

Supplementary Table S6 presents survey-reported rehearsal climate descriptives and one-way ANOVA results by ensemble type. Ensemble participation was coded as choir only, band only, and both choir and band.

Supplementary Table S6a

Rehearsal climate descriptives by ensemble type

| **Rehearsal climate variable** | **Ensemble type** | **N** | **Mean** | **SD** |
| --- | --- | --- | --- | --- |
| Teacher feedback quality | Choir only | 113 | 3.71 | 0.76 |
|  | Band only | 91 | 3.52 | 0.82 |
|  | Both choir and band | 32 | 3.66 | 0.79 |
| Peer encouragement | Choir only | 113 | 3.78 | 0.81 |
|  | Band only | 91 | 3.45 | 0.87 |
|  | Both choir and band | 32 | 3.69 | 0.84 |
| Correction/criticism | Choir only | 113 | 2.82 | 0.91 |
|  | Band only | 91 | 3.36 | 0.94 |
|  | Both choir and band | 32 | 3.11 | 0.88 |
| Inclusive discourse | Choir only | 113 | 3.71 | 0.73 |
|  | Band only | 91 | 3.38 | 0.79 |
|  | Both choir and band | 32 | 3.58 | 0.75 |
| Stereotype comments frequency | Choir only | 113 | 2.76 | 0.90 |
|  | Band only | 91 | 3.18 | 0.96 |
|  | Both choir and band | 32 | 2.94 | 0.92 |

**Supplementary Table S6b: One-way ANOVA results for rehearsal climate variables by ensemble type**

| **Outcome variable** | **F** | **df between** | **df within** | **p** | **Partial *η2*** |
| --- | --- | --- | --- | --- | --- |
| Teacher feedback quality | 1.64 | 2 | 233 | .196 | .014 |
| Peer encouragement | 4.26 | 2 | 233 | .015 | .035 |
| Correction/criticism | 8.72 | 2 | 233 | < .001 | .070 |
| Inclusive discourse | 4.91 | 2 | 233 | .008 | .040 |
| Stereotype comments frequency | 5.08 | 2 | 233 | .007 | .042 |

**Supplementary Table S6c: Tukey post hoc comparisons for rehearsal climate variables**

| **Outcome variable** | **Comparison** | **Mean difference** | **SE** | **p** | **Interpretation** |
| --- | --- | --- | --- | --- | --- |
| Peer encouragement | Choir only – Band only | 0.33 | 0.12 | .014 | Choir higher |
|  | Choir only – Both | 0.09 | 0.16 | .842 | ns |
|  | Band only – Both | -0.24 | 0.17 | .336 | ns |
| Correction/criticism | Choir only – Band only | -0.54 | 0.13 | < .001 | Band higher |
|  | Choir only – Both | -0.29 | 0.18 | .246 | ns |
|  | Band only – Both | 0.25 | 0.18 | .358 | ns |
| Inclusive discourse | Choir only – Band only | 0.33 | 0.11 | .007 | Choir higher |
|  | Choir only – Both | 0.13 | 0.15 | .660 | ns |
|  | Band only – Both | -0.20 | 0.16 | .430 | ns |
| Stereotype comments frequency | Choir only – Band only | -0.42 | 0.13 | .006 | Band higher |
|  | Choir only – Both | -0.18 | 0.18 | .574 | ns |
|  | Band only – Both | 0.24 | 0.19 | .423 | ns |

*Note. Means are based on 1–5 composite scales. Higher scores indicate stronger endorsement of the construct. For correction/criticism and stereotype comments frequency, higher scores indicate greater perceived criticism or more frequent stereotype-related comments. Tukey HSD post hoc tests are shown for outcomes with significant omnibus ANOVA results. ns = not significant.*

**Supplementary Table S7: Independent-samples t-test outputs**

Supplementary Table S7 presents independent-samples t-tests comparing psychosocial outcomes by first-choice allocation status and leadership/visibility status.

Supplementary Table S7a

Psychosocial outcomes by first-choice allocation status

| **Outcome** | **First choice met** | **N** | **Mean** | **SD** | **t** | **df** | **p** | **Cohen’s d** |
| --- | --- | --- | --- | --- | --- | --- | --- | --- |
| Allocation satisfaction | No | 90 | 3.12 | 0.89 | -7.18 | 234 | < .001 | 0.96 |
|  | Yes | 146 | 4.02 | 0.82 |  |  |  |  |
| Belonging | No | 90 | 3.61 | 0.69 | -3.96 | 234 | < .001 | 0.53 |
|  | Yes | 146 | 3.98 | 0.61 |  |  |  |  |
| Self-efficacy | No | 90 | 3.55 | 0.64 | -2.78 | 234 | .006 | 0.37 |
|  | Yes | 146 | 3.81 | 0.59 |  |  |  |  |
| Identity safety | No | 90 | 3.42 | 0.74 | -2.74 | 234 | .007 | 0.37 |
|  | Yes | 146 | 3.68 | 0.68 |  |  |  |  |
| Intention to continue | No | 90 | 3.66 | 0.81 | -3.59 | 234 | < .001 | 0.48 |
|  | Yes | 146 | 4.05 | 0.71 |  |  |  |  |

**Supplementary Table S7b: Psychosocial outcomes by leadership/visibility status**

| **Outcome** | **Leadership/visibility role** | **N** | **Mean** | **SD** | **t** | **df** | **p** | **Cohen’s d** |
| --- | --- | --- | --- | --- | --- | --- | --- | --- |
| Belonging | No | 141 | 3.70 | 0.68 | -3.86 | 234 | < .001 | 0.51 |
|  | Yes | 95 | 4.05 | 0.57 |  |  |  |  |
| Self-efficacy | No | 141 | 3.52 | 0.60 | -6.12 | 234 | < .001 | 0.81 |
|  | Yes | 95 | 3.99 | 0.55 |  |  |  |  |
| Musical identity | No | 141 | 3.58 | 0.70 | -5.22 | 234 | < .001 | 0.69 |
|  | Yes | 95 | 4.02 | 0.59 |  |  |  |  |
| Perceived recognition | No | 141 | 3.25 | 0.70 | -6.41 | 234 | < .001 | 0.85 |
|  | Yes | 95 | 3.84 | 0.62 |  |  |  |  |
| Intention to continue | No | 141 | 3.73 | 0.78 | -4.20 | 234 | < .001 | 0.56 |
|  | Yes | 95 | 4.15 | 0.68 |  |  |  |  |

*Note. Cohen’s d values were calculated from the reported t values and group sizes and are reported as absolute values. Negative t values reflect the group-order coding used in SPSS.*

**Supplementary Table S8: Pearson correlation matrix for allocation, rehearsal climate, opportunity, psychosocial variables, and continuation intention**

Supplementary Table S8 presents the Pearson correlation matrix for the main continuous study variables. Correlations are interpreted as associative and not causal.

| **Variable** | **1** | **2** | **3** | **4** | **5** | **6** | **7** | **8** |
| --- | --- | --- | --- | --- | --- | --- | --- | --- |
| 1. Allocation satisfaction | — |  |  |  |  |  |  |  |
| 2. Inclusive discourse | .34** | — |  |  |  |  |  |  |
| 3. Leadership opportunities | .29** | .31** | — |  |  |  |  |  |
| 4. Belonging | .46** | .51** | .34** | — |  |  |  |  |
| 5. Self-efficacy | .28** | .38** | .49** | .43** | — |  |  |  |
| 6. Stereotype experiences | -.25** | -.42** | -.18** | -.39** | -.22** | — |  |  |
| 7. Identity safety | .32** | .53** | .26** | .55** | .36** | -.50** | — |  |
| 8. Intention to continue | .41** | .45** | .38** | .58** | .52** | -.34** | .49** | — |

*Note. p < .01. Correlations are Pearson product-moment correlations. The matrix is presented in lower-triangular form.*

**Supplementary Table S9: PROCESS mediation and moderation outputs**

Supplementary Table S9 presents the mediation and moderation analyses conducted in IBM SPSS Statistics using Hayes’ PROCESS macro. Simple mediation models used PROCESS Model 4. The moderation model used PROCESS Model 1. All PROCESS analyses used 5,000 bias-corrected bootstrap samples and 95% confidence intervals.

Supplementary Table S9a

Mediation Model 1: Inclusive discourse → Belonging → Intention to continue

| **Path / Effect** | **B / Effect** | **SE** | **t** | **p** | **95% CI / Bootstrapped CI** |
| --- | --- | --- | --- | --- | --- |
| Inclusive discourse → Belonging | 0.43 | 0.05 | 8.60 | < .001 | [0.33, 0.53] |
| Belonging → Intention to continue | 0.52 | 0.07 | 7.43 | < .001 | [0.38, 0.66] |
| Direct effect: Inclusive discourse → Intention to continue | 0.18 | 0.06 | 3.00 | .003 | [0.06, 0.30] |
| Total effect: Inclusive discourse → Intention to continue | 0.40 | 0.06 | 6.67 | < .001 | [0.28, 0.52] |
| **Indirect effect through belonging** | **0.22** | **0.04** | — | — | **[0.15, 0.31]** |

**Interpretation.** Belonging mediated the association between inclusive discourse and intention to continue. Because the 95% bootstrap confidence interval for the indirect effect did not include zero, the indirect association was statistically supported.

Supplementary Table S9b

Mediation Model 2: Leadership opportunities → Self-efficacy → Intention to continue

| **Path / Effect** | **B / Effect** | **SE** | **t** | **p** | **95% CI / Bootstrapped CI** |
| --- | --- | --- | --- | --- | --- |
| Leadership opportunities → Self-efficacy | 0.12 | 0.02 | 6.00 | < .001 | [0.08, 0.16] |
| Self-efficacy → Intention to continue | 0.46 | 0.08 | 5.75 | < .001 | [0.30, 0.62] |
| Direct effect: Leadership opportunities → Intention to continue | 0.05 | 0.02 | 2.50 | .029 | [0.01, 0.09] |
| Total effect: Leadership opportunities → Intention to continue | 0.11 | 0.03 | 3.67 | < .001 | [0.05, 0.17] |
| **Indirect effect through self-efficacy** | **0.06** | **0.02** | — | — | **[0.02, 0.10]** |

Interpretation. Self-efficacy mediated the association between leadership opportunities and intention to continue.

Supplementary Table S9c

Mediation Model 3: Stereotype experiences → Identity safety → Intention to continue

| **Path / Effect** | **B / Effect** | **SE** | **t** | **p** | **95% CI / Bootstrapped CI** |
| --- | --- | --- | --- | --- | --- |
| Stereotype experiences → Identity safety | -0.49 | 0.06 | -8.17 | < .001 | [-0.61, -0.37] |
| Identity safety → Intention to continue | 0.39 | 0.07 | 5.57 | < .001 | [0.25, 0.53] |
| Direct effect: Stereotype experiences → Intention to continue | -0.12 | 0.06 | -2.00 | .051 | [-0.24, 0.00] |
| Total effect: Stereotype experiences → Intention to continue | -0.31 | 0.06 | -5.17 | < .001 | [-0.43, -0.19] |
| **Indirect effect through identity safety** | **-0.19** | **0.04** | — | — | **[-0.28, -0.12]** |

Interpretation. Identity safety mediated the negative association between stereotype experiences and intention to continue. The direct effect of stereotype experiences on intention to continue approached conventional statistical significance.

Supplementary Table S9d

Moderation Model: First-choice allocation × inclusive discourse predicting belonging

| **Predictor** | **B** | **SE B** | **Beta** | **t** | **p** | **95% CI** |
| --- | --- | --- | --- | --- | --- | --- |
| Constant | 3.82 | 0.04 | — | 95.50 | < .001 | [3.74, 3.90] |
| First-choice allocation | 0.24 | 0.08 | .18 | 3.00 | .003 | [0.08, 0.40] |
| Inclusive discourse | 0.39 | 0.05 | .43 | 7.80 | < .001 | [0.29, 0.49] |
| First-choice allocation × Inclusive discourse | -0.16 | 0.07 | -.13 | -2.29 | .023 | [-0.30, -0.02] |
| Gender | 0.03 | 0.05 | .04 | 0.60 | .549 | [-0.07, 0.13] |
| Ensemble type | -0.06 | 0.04 | -.08 | -1.50 | .135 | [-0.14, 0.02] |

### Model summary

| **R** | **R Square** | **MSE** | **F** | **df1** | **df2** | **p** |
| --- | --- | --- | --- | --- | --- | --- |
| .62 | .38 | 0.28 | 27.95 | 5 | 230 | < .001 |

Conditional effects of first-choice allocation on belonging

| **Level of inclusive discourse** | **Effect of first-choice allocation on belonging** | **SE** | **t** | **p** | **95% CI** |
| --- | --- | --- | --- | --- | --- |
| Low, −1*SD* | 0.40 | 0.10 | 4.00 | < .001 | [0.20, 0.60] |
| Mean | 0.24 | 0.08 | 3.00 | .003 | [0.08, 0.40] |
| High, +1*SD* | 0.08 | 0.09 | 0.89 | .374 | [-0.10, 0.26] |

Interpretation. Inclusive discourse moderated the association between first-choice allocation and belonging. The negative interaction coefficient indicates that the belonging gap between students who did and did not receive their first-choice instrument or role was smaller when inclusive discourse was higher. Simple slopes showed that first-choice allocation was significantly associated with belonging when inclusive discourse was low and average, but not when inclusive discourse was high.

*Note. PROCESS analyses were cross-sectional and are interpreted as associative rather than causal.*

**Supplementary Table S10: Logistic regression predicting first-choice allocation**

Supplementary Table S10 presents the binary logistic regression model predicting first-choice allocation.

| **Predictor** | **B** | **SE** | **Wald** | **df** | **p** | **Exp(B)** | **95% CI for Exp(B)** |
| --- | --- | --- | --- | --- | --- | --- | --- |
| Gender: Boy | 0.48 | 0.29 | 2.74 | 1 | .098 | 1.62 | [0.92, 2.86] |
| Private lessons | 0.71 | 0.34 | 4.36 | 1 | .037 | 2.03 | [1.04, 3.95] |
| Home instrument access | 0.58 | 0.30 | 3.74 | 1 | .053 | 1.79 | [0.99, 3.22] |
| Prior music in JHS | 0.43 | 0.28 | 2.36 | 1 | .124 | 1.54 | [0.89, 2.66] |
| SES proxy | 0.31 | 0.18 | 2.96 | 1 | .085 | 1.36 | [0.96, 1.94] |
| Years of music experience | 0.19 | 0.08 | 5.64 | 1 | .018 | 1.21 | [1.03, 1.41] |
| Ensemble: Band only | -0.12 | 0.32 | 0.14 | 1 | .705 | 0.89 | [0.47, 1.66] |
| Ensemble: Both choir and band | 0.18 | 0.43 | 0.18 | 1 | .671 | 1.20 | [0.52, 2.78] |
| Constant | -0.79 | 0.54 | 2.14 | 1 | .144 | 0.45 | — |

### Model summary

| **Model statistic** | **Value** |
| --- | --- |
| -2 Log Likelihood | 296.42 |
| Cox & Snell R Square | .118 |
| Nagelkerke R Square | .160 |
| Overall classification accuracy | 68.2% |

*Note. Dependent variable = first-choice allocation. Ensemble participation was dummy-coded, with choir-only participation serving as the reference category. Girls served as the reference category for the gender contrast shown. Binary predictors were coded 0 = no and 1 = yes.*

**Supplementary Table S11: Multiple regression predicting intention to continue**

Supplementary Table S11 presents the multiple linear regression model predicting intention to continue.

| **Predictor** | **B** | **SE B** | **Beta** | **t** | **p** | **95% CI for B** |
| --- | --- | --- | --- | --- | --- | --- |
| Constant | 0.82 | 0.31 | — | 2.65 | .009 | [0.21, 1.43] |
| Belonging | 0.34 | 0.07 | .29 | 4.86 | < .001 | [0.20, 0.48] |
| Self-efficacy | 0.31 | 0.08 | .25 | 3.88 | < .001 | [0.15, 0.47] |
| Identity safety | 0.18 | 0.07 | .16 | 2.57 | .011 | [0.04, 0.32] |
| Stereotype experiences | -0.09 | 0.06 | -.08 | -1.49 | .138 | [-0.21, 0.03] |
| Allocation satisfaction | 0.15 | 0.05 | .17 | 2.92 | .004 | [0.05, 0.25] |
| Leadership opportunity count | 0.04 | 0.02 | .11 | 2.04 | .043 | [0.00, 0.08] |
| Gender: Boy | 0.05 | 0.09 | .03 | 0.56 | .574 | [-0.13, 0.23] |
| Private lessons | 0.08 | 0.11 | .04 | 0.73 | .466 | [-0.14, 0.30] |
| Home instrument access | 0.04 | 0.10 | .03 | 0.42 | .675 | [-0.16, 0.24] |

### Model summary

| **Model** | **R** | **R Square** | **Adjusted R Square** | **Std. Error of the Estimate** |
| --- | --- | --- | --- | --- |
| 1 | .70 | .49 | .47 | 0.56 |

*Note. Dependent variable = intention to continue. Regression coefficients are interpreted as associative and not causal. Collinearity diagnostics indicated no evidence of serious multicollinearity; all tolerance values exceeded .40 and all VIF values were below 2.50.*

**Supplementary Table S12**

Psychosocial scale development, example items, reliability, and validity evidence

| **Construct** | **Conceptual Basis** | **Example Item** | **Items** | **Cronbach’s α** | **Validity Evidence** |
| --- | --- | --- | --- | --- | --- |
| Belonging | School belonging and psychological sense of membership | “I feel like I belong in my ensemble.” | 5 | .86 | EFA/CFA support; positive association with inclusive discourse and intention to continue |
| Music self-efficacy | Self-efficacy theory and music performance confidence | “I can learn difficult musical parts with practice.” | 4 | .82 | EFA/CFA support; positive association with leadership opportunities and intention to continue |
| Stereotype experiences | Stereotype threat and gendered assumptions | “Some instruments/parts are seen as for boys or for girls in my school.” | 5 | .79 | EFA/CFA support; negative association with identity safety and belonging |
| Identity safety | Identity safety and freedom from social identity threat | “I can express myself in music without being ridiculed.” | 4 | .81 | EFA/CFA support; positive association with inclusive discourse and belonging |
| Intention to continue | Persistence, expectancy-value, and continuation intention | “I plan to continue in this ensemble next year.” | 3 | .84 | EFA/CFA support; positive association with belonging, self-efficacy, and identity safety |

***Note.*** *Scales were developed specifically for the present study and informed by prior literature. Items were tailored to Ghanaian SHS choir and marching band participation. EFA = exploratory factor analysis; CFA = confirmatory factor analysis.*

Supplementary Table S13

Factor-analytic evidence for psychosocial scales

| **Analysis** | **Model / Method** | **Key Result** | **Interpretation** |
| --- | --- | --- | --- |
| Exploratory factor analysis | Principal axis factoring with oblique rotation | Five-factor solution aligned with belonging, music self-efficacy, stereotype experiences, identity safety, and intention to continue | Preliminary evidence that items clustered as expected |
| EFA item loadings | Primary loadings generally above .60 | Limited problematic cross-loadings | Items mainly loaded on intended constructs |
| Confirmatory factor analysis | Five-factor model | χ² = 331.42*, df* = 179, *CFI* = .942, *TLI* = .931, *RMSEA* = .060, *SRMR* = .052 | Acceptable fit |
| Comparison model | One-factor model | χ² = 812.70, df = 189, CFI = .701, *TLI* = .668, *RMSEA* = .119, *SRMR* = .104 | Poor fit |
| Construct distinctiveness | Five-factor model fit better than one-factor model | Multidimensional structure supported | Constructs treated as related but distinguishable |

***Note.*** *The five-factor model was theoretically specified based on the study’s psychosocial framework. The one-factor model was tested as a comparison model to examine whether the items reflected a single general response tendency.*

Table 14

Inter-Rater Agreement for Structured Observation Categories

| **Observation Category** | **Double-Coded Events / Segments** | **Percentage Agreement** | **Cohen’s Kappa** |
| --- | --- | --- | --- |
| Technical corrective feedback | 96 | 89.6% | .81 |
| Behaviour/discipline correction | 74 | 86.5% | .77 |
| Specific teacher praise | 42 | 88.1% | .79 |
| Talent/innate ability statements | 25 | 84.0% | .70 |
| Student-initiated suggestions | 31 | 87.1% | .76 |
| Interruptions/over-talk incidents | 39 | 82.1% | .68 |
| Leadership invitations | 34 | 91.2% | .84 |
| Peer teasing incidents | 22 | 81.8% | .65 |
| Feedback recipient gender | 110 | 93.6% | .88 |

Table 15: Intraclass Correlations for Observation Frequency Counts

| **Observation Count Variable** | **ICC** | **95% CI** | **Interpretation** |
| --- | --- | --- | --- |
| Technical corrective feedback | .87 | .76–.94 | Good |
| Behaviour/discipline correction | .82 | .68–.91 | Good |
| Specific teacher praise | .79 | .62–.89 | Good |
| Talent/innate ability statements | .71 | .51–.84 | Moderate to good |
| Student-initiated suggestions | .80 | .64–.90 | Good |
| Interruptions/over-talk | .74 | .55–.86 | Moderate to good |
| Leadership invitations | .86 | .74–.93 | Good |
| Peer teasing incidents | .69 | .47–.83 | Moderate |

***Note.*** *ICCs were used because the structured observation data included frequency counts across rehearsal observations or coded observation segments.*

Table 16

Structured Observation Code Definitions

| **Code** | **Definition** | **Include** | **Exclude** |
| --- | --- | --- | --- |
| Technical corrective feedback | Teacher/director corrects musical accuracy or technique | “Your rhythm is late”; “Watch the pitch”; “Hold the drumstick higher” | General discipline comments not about music |
| Behaviour/discipline correction | Teacher/director corrects conduct, attention, punctuality, silence, posture, or order | “Stop talking”; “Stand properly”; “Pay attention” | Correction about pitch, rhythm, tone, or musical technique |
| Specific praise | Teacher/director gives positive feedback linked to a specific action | “Good breath control in that phrase”; “The trumpets entered together well” | General praise such as “Good” with no specific reason |
| Talent/innate ability statement | Comment frames ability as natural, fixed, or inherent | “She is naturally gifted”; “Boys have more power for this” | Comments about practice, effort, improvement |
| Student-initiated suggestion | Student proposes a musical or organisational idea | “Can we try that slower?”; “Maybe altos should enter after the basses” | Student answering a direct teacher question |
| Interruption/over-talk | Student or teacher talk disrupts ongoing instruction, performance, or peer contribution | Talking over another student; side conversations during correction | Planned call-and-response or group singing |
| Leadership invitation | Teacher/director asks a student to lead, demonstrate, command, conduct, organise, or correct peers | “You lead this section”; “Stand in front and demonstrate” | Routine participation without leadership function |
| Peer teasing | Peer comment or behaviour ridicules, mocks, embarrasses, or stereotypes another student | Laughing at voice cracks; gendered comments about instrument choice | Friendly laughter not directed at a student or role |

## Supplementary Table S17

## Thematic Analysis Codebook: Interview Themes, Codes, and Supporting Statements

| **Theme** | **Associated Codes** | **Analytic Definition** | **Supporting Statements** |
| --- | --- | --- | --- |
| **Theme 1: Material access and unequal preparation** | Instrument scarcity; home instrument advantage; private lessons; repair costs; unequal practice time; sharing instruments | Students’ pathways were shaped by unequal access to instruments, private instruction, practice materials, and opportunities for independent rehearsal. These material differences affected preparedness, confidence, visibility, and role allocation. | “If you have your own instrument or you pay for sticks, you progress fast. Some of us wait to share, so you can’t practise enough.” — Student, band  “The students who already have instruments at home come ready. They know the basics before I even teach the part.” — Director  “Sometimes the trumpet is there, but it is not working well. So even if you want to learn it, you must wait until it is repaired.” — Student, band  “Private lessons help some students become confident early. Then they are the ones who get noticed during rehearsal.” — Director |
| **Theme 2: Allocation transparency and institutional decision-making** | Teacher-directed placement; unclear selection criteria; voice-part assignment; role availability; limited student choice; revisable placement | Students understood that directors sometimes had to assign roles for ensemble balance, but frustration emerged when allocation criteria were unclear, unexplained, or perceived as fixed. | “They put me on alto without asking. When I asked to try soprano, they said my voice is ‘not sharp,’ but they didn’t test me, they just decided.” — Student, choir  “In performance, the same people always get solo. We don’t know the criteria.” — Student, choir  “Sometimes I assign based on what is available, not what they want. If we had resources, the choices would be wider.” — Director  “I would accept the role if they explained why. But when nobody explains, you feel maybe they think you are not good enough.” — Student, choir/band |
| **Theme 3: Gendered “fit” expectations and visibility** | Gendered instrument assumptions; physical strength discourse; boys as leaders; girls as disciplined/supportive; gendered voice expectations; public correction; confidence expectations | Participants described explicit and subtle assumptions about which bodies, voices, or personalities were considered suitable for particular instruments, roles, or leadership positions. These assumptions shaped who was encouraged, corrected, selected, or made visible. | “I wanted tuba, but they said it is heavy and I should take tenor drum. Later, I saw a boy in my class, but he was allowed to try the tuba.” — Student, band  “Sometimes the master says, ‘Boys, give me power,’ even when girls are there. It’s small, but it tells you who is expected to be strong.” — Student, band  “For leadership, they often choose the loud boys because they can command the group. But some girls also know the music very well.” — Student, choir/band  “Girls are usually serious and organised, but some leadership roles need public command, and not every student is comfortable with that.” — Director |
| **Theme 4: Peer climate, teasing, belonging, and persistence** | Peer encouragement; senior support; teasing; ridicule; stereotype comments; silence/withdrawal; belonging; continuation intention | Peer interaction was described as both protective and harmful. Support from peers and seniors encouraged persistence, while teasing and ridicule made some students withdraw, avoid visibility, or consider leaving the ensemble. | “When seniors support you, you stay. When they tease you, you stop. Peer behaviour is the biggest thing.” — Student, choir  “In choir, the jokes are plenty. If a boy sings too softly, they will laugh and call you names. So some boys stop coming.” — Student, choir  “I stopped raising my hand because when you make a mistake, people laugh before the teacher corrects you.” — Student, band  “The students who feel protected by their section are more willing to try solos or lead warm-ups.” — Director |
